# Supplementary material for: Price elasticity of demand for cigarettes in Nepal: Evidence from a lower middle-income country in South Asia using Deaton’s demand model
Source: PLoS One. 2026 Mar 30;21(3):e0345594. doi: 10.1371/journal.pone.0345594 (PMC13035141; doi:10.1371/journal.pone.0345594)
Supplement: Attachment — Submitted filename: PONE-D-25-25792_R1_reviewer-1.pdf [file pone.0345594.s003.pdf]

PLOS ONE

Price elasticity of demand for cigarettes in Nepal: evidence from a lower middle-income country in South Asia using Deaton's demand model

--Manuscript Draft--

**Manuscript Number:**	PONE-D-25-25792R1
**Article Type:**	Research Article
**Full Title:**	Price elasticity of demand for cigarettes in Nepal: evidence from a lower middle-income country in South Asia using Deaton's demand model
**Short Title:**	Price elasticity of demand for cigarettes in Nepal
**Corresponding Author:**	Vishnu Prasad Sapkota, M.Phil. Economics; M.Sc. Public Health<br>Tribhuvan University Nepal Commerce Campus<br>Kathmandu, Bagmati NEPAL
**Keywords:**	Price elasticity; Cigarette consumption; Taxation; tobacco industry; Tobacco policy
**Abstract:**	Background: The prevalence of cigarette smoking is declining globally; however, it remains highest in South Asia. Governments in this region rely on taxation as an instrument to reduce cigarette demand. The effectiveness of such instruments depends on consumers' underlying preferences and behavioural responses to changes in price – it is commonly expressed in terms of price elasticity of demand. A few studies in this region used available cross-sectional and panel surveys to estimate cigarette price elasticity using Deaton's demand model. In this context, we aim to estimate the price elasticity of cigarettes in Nepal.<br>Methods: We applied Deaton's demand model to estimate the cigarette price elasticity using a three-year panel microdata (2016-2018) from the Nepal Household Risk and Vulnerability Survey. We used data from 5,653 households observed over three waves; the study units reported the purchased quantities of cigarettes and related expenditures. Deaton's demand model exploits spatial variation in unit values of cigarettes to measure price elasticity. We adjusted the basic model for possible bias that may arise from panel nature of the data.<br>Results: We found that the estimate of price elasticity for cigarette demand was -0.58 with [95% CI: -0.79 -0.37], and the expenditure elasticity estimate was 1.06 [95% CI: 0.54 – 1.57]. These statistics indicate that the cigarette demand is relatively inelastic to price, and unitary elastic to income.<br>Conclusions: The negative price elasticity estimate for Nepal indicates that the increase in excise taxes can be effective both in reducing cigarette consumption and raising revenues. This also provides additional evidence that the routine surveys, including panel data, that are designed to monitor living standards can be valuable to estimate price elasticity using the Deaton's method.
**Order of Authors:**	Vishnu Prasad Sapkota, M.Phil. Economics; M.Sc. Public Health
	Umesh Prasad Bhusal, Masters of Public Health (Health Economics)
	Nirmal Kumar Raut, PhD-Economics
**Response to Reviewers:**	10/10/2025<br><br>To<br><br>Gulcan Onel, Ph.D.<br>Academic Editor<br>PLOS ONE<br><br>Dear Editor,<br>RE: PONE-D-25-25792 – Price elasticity of demand for cigarettes in Nepal: evidence from a lower middle-income country in South Asia using Deaton's demand model<br>Many thanks for considering our paper and for giving us the opportunity to resubmit after addressing the reviewers' valuable comments. We thank the reviewers for reading the paper carefully and providing comments that have been tremendously helpful in improving it. We include a document that gives a point-by-point response to

the comments. We believe that we have addressed all of them.
Please do ask if we have missed anything. We look forward to receiving your decision.
Yours sincerely,

Vishnu Prasad Sapkota
Lecturer
Department of Economics, Tribhuvan University, Nepal Commerce Campus
New Baneshwor, Kathmandu, Nepal
vishnu.sapkota@ncc.tu.edu.np

We thank the reviewers for reading our paper carefully and providing valuable comments that have been tremendously helpful in improving it. This document explains our response to each comment.

Reviewer #1:

Comment 1: The introduction is lengthy and could be more concise, particularly in discussing regional comparisons. Streamline the introduction by condensing regional comparisons into a single paragraph.

Response: Thank you very much for this feedback. We have reviewed the Introduction section carefully and made it more concise. We have condensed the regional comparisons. The changes can be found in the tracked-changes version of the revised manuscript.

Comment 2: There is need to provide an overview of the trend of tobacco prices and/or excise tax in Nepal. This should be part of the Introduction section.

Response: Thank you again for this important suggestion. We have added the following detail in the fourth paragraph of the introduction section.

Table 1 below shows the trend of cigarette prices and taxes in Nepal over the period of 10 years. The cigarette prices have been increasing at constant rate of ~1.00 USD (in PPP) between 2012 to 2022. This means that the household face similar increment in prices before and during the survey period. During the same period, the excise taxes have remained stable between 26 – 30 during the survey period.

Table 1: Cigarette price and tax in Nepal between 2012 and 2022.
201220142016201820202022
Retain price of cigarette per 20 sticks in PPP dollars3.654.695.636.788.3810.43
Taxes as percentage of cigarette price (%)29.8627.7926302731.39
Source: https://www.who.int/data/gho/data/themes/topics/raise-taxes-on-tobacco

Comment 3: The numerator for Equation 7 should have a w bar, not an underlined w.

Response: Thank you. We have corrected this.

Comment 4: The discussion is lengthy and repetitive, particularly in comparing expenditure shares and coefficients across countries. Condense the comparison of expenditure shares and coefficients into a table for clarity and brevity.

Response: Thank you for providing opportunity to revise the discussion. We have removed the repetitive points in the discussion and attempted to make the comparison condense. We have revised the section as follows.

The findings related to prevalence and expenditure patterns using data from Nepal exhibit consistency when compared with its South Asian Counterparts as well as other LMICs. While Nepalese households that incur non-zero spending are 15 percent, which is higher than the regional average of 10 percent, the statistics lie within the range from 4.5 percent in Bhutan to 18.7 percent in the Maldives (19). The prevalence reported elsewhere exhibits wide variation, ranging from 1.5 percent in Nigeria to 7 percent in Uganda and 40 percent in Bosnia and Herzegovina and Serbia (28, 29, 31-33). Expenditure shares (5.14 percent for Nepal), on the other hand, exhibit limited variation, from 3.9 percent in India to 7.6 percent in Uganda (18, 29, 33, 34). Likewise, the regression estimates for unit value (0.78) and budget share (0.0066) also fall within the wide range reported in the literature, which varies across the member nations in the region and other LMICs (14, 17, 28, 29, 31, 33, 35, 36). So far as price elasticity of demand is concerned, global evidence suggests wide variation, exhibiting both elastic and inelastic demands. For example, Vietnam (-0.165) and Zambia (-0.2) exhibit inelastic demand, while Pakistan (-1.06), rural Bangladesh (-1.38), and Bosnia and Herzegovina (-1.366) exhibit elastic demand. Most studies generally find inelastic demand for cigarettes (17, 33, 37, 39, 40, 41), but there is substantial cross-country

Powered by Editorial Manager® and ProduXion Manager® from Aries Systems Corporation

variation in how smokers respond to changes in the price of cigarettes.
Comment 5: No mention of future research directions beyond the current limitations.
Response: As suggested, we have identified areas of future research (See Discussion, Paragraphs 5) by adding following paragraph.
Depending upon the data availability, it may be possible to extend this study, where researchers may consider heterogeneity of the sample population for analysis (for example, based on income levels, rural-urban settings, age groups, and gender). This has the advantage of making tax and tobacco control policies more targeted and fairer. It is also plausible to collect data at frequent intervals and examine the smokers' behaviour, since this will help make informed and timely policy and behaviour-related decisions. Likewise, new surveys available in the region comprise a broader set of questions related to tobacco and cigarette smoking. It may be meaningful to utilize the datasets from these surveys to examine and make inter- and intra-regional comparisons of the shifts in the behavioural patterns of the smokers.
Comment 6: The referencing should be reviewed. For instance, Walker et al. (2019)(27) should be Walker et al. (27). Also, note that there has to be space before an open bracket – review the whole manuscript.
Response: Thank you. We have now corrected the references.

Reviewer #2:
Comment 1: To be more specific Deaton's model is well suited for the cross-sectional data analysis and independent household observations. There seem to be some efforts from authors in this domain however are not well described and thus not possible to assess in the terms of their appropriateness and violating the main feature of current research in terms of replicability.
Response: Thank you very much for providing us with an opportunity to elaborate on how the extension of Deaton's approach to panel data does provide useful results after reasonable assumptions and suitable adjustments. In the methodology section (specification and estimation, paragraph 3), we have added the following paragraphs.
In the pooled OLS set-up of equations 1 and 2, we adjust for possible bias that may arise from repeated measurements over three waves, possibly due to temporal trends in unit values and budget shares, temporal correlation in the error terms of the respective equations, and unobserved changes in cigarette consumption patterns that may be correlated with the covariates. We correct for these as follows:
We add year-specific fixed effects to ensure that between-cluster variation in the unit value and budget share regressions is free from temporal trends. This ensures that the basic requirement to apply the Deaton demand model is maintained.
In these equations, within-cluster variation may be due to (i) variation in cigarette consumption preferences over the three-year period, (ii) autocorrelation in the error terms in equations 1 and 2, and (iii) measurement error (correlation between the error terms of equations 1 and 2). Of these, (iii) is key to Deaton's correction strategy for measurement error. We minimize bias that may result from (i) and (ii) with the following adjustments and assumptions:
We adjust for temporal correlation in u_hct^1and u_hct^oby allowing the errors to cluster over time while estimating the measurement error.
We assume that policy-driven shifts in cigarette preferences are minimal during the survey period – a stable, marginal change in cigarette prices and taxes between 2016–2018 (shown in Table 1) provides evidence that this assumption is met.
Cigarette consumption behaviour exhibits a stable pattern over short time interval. A one-year gap between each survey wave makes preference changes unlikely – longitudinal studies show that transitions between smoker and non-smoker states are rare within one year (24).
Together, with these assumptions and adjustments, the coefficient estimates from the pooled OLS and measurement error corrections still maintain the properties of the original Deaton (1988) model.

Comment 2: For robustness of the results, it would be preferable to use also all three waves of surveys to estimate elasticities separately and present results for those as further validation of the approach.
Response: Yes, we have performed robustness checks in individual years. The estimate ranges -0.15 in 2016 to -0.95 in 2018, and both values indicate inelastic demand. These values are reported in the notes of Table 5 as follows.
We also undertake robustness checks of (ε_p)̂ across the three survey panels – the estimates range from -0.15 in 2016 to -0.95 in 2018.

Powered by Editorial Manager® and ProduXion Manager® from Aries Systems Corporation

	Comment 3: please do a thorough revision of the literature review as some of the information seem not to fit the contents of the papers cited. For instance, reference (14) to John (2008) indicate that for additional products to bidis and leaf tobacco elasticities are estimated, however the paper only investigates cigarettes, bidis and leaf tobacco.<br>Response: Thank you very much for providing an opportunity to revise our literature review and discussion. We have thoroughly revised it, and it is available in the track changes in the text.
**Additional Information:**	
**Question**	**Response**
**Financial Disclosure**<br><br>Enter a financial disclosure statement that describes the sources of funding for the work included in this submission. Review the submission guidelines for detailed requirements. View published research articles from *PLOS ONE* for specific examples.<br><br>This statement is required for submission and **will appear in the published article** if the submission is accepted. Please make sure it is accurate.<br><br>**Funded studies**<br>Enter a statement with the following details:<br>• Initials of the authors who received each award<br>• Grant numbers awarded to each author<br>• The full name of each funder<br>• URL of each funder website<br>• Did the sponsors or funders play any role in the study design, data collection and analysis, decision to publish, or preparation of the manuscript?<br><br>Did you receive funding for this work?	The author(s) received no specific funding for this work.
**Competing Interests**<br><br>Use the instructions below to enter a competing interest statement for this submission. On behalf of all authors, disclose any competing interests that could be perceived to bias this work—acknowledging all financial support and any other relevant financial or non-financial competing interests.	The authors have declared that no competing interests exist.

This statement is required for submission and **will appear in the published article** if the submission is accepted. Please make sure it is accurate and that any funding sources listed in your Funding Information later in the submission form are also declared in your Financial Disclosure statement.<br><br>View published research articles from *PLOS ONE* for specific examples.<br><br>**NO authors have competing interests**<br><br>Enter: *The authors have declared that no competing interests exist.*<br><br>**Authors with competing interests**<br><br>Enter competing interest details beginning with this statement:<br><br>*I have read the journal's policy and the authors of this manuscript have the following competing interests: [insert competing interests here]*<br><br>* typeset	
**Ethics Statement**<br><br>Enter an ethics statement for this submission. This statement is required if the study involved:<br><br>• Human participants<br>• Human specimens or tissue<br>• Vertebrate animals or cephalopods<br>• Vertebrate embryos or tissues<br>• Field research<br><br>Write "N/A" if the submission does not require an ethics statement.<br><br>General guidance is provided below. Consult the submission guidelines for detailed instructions. **Make sure that all information entered here is included in the**	The analysis is based on the survey data that was conducted based on the protocol for the survey was approved by the Central Bureau of Statistics (CBS) as per the Statistical Act (1958) in September 2018. Verbal consent was obtained from each respondent after a thorough introduction of the survey. All respondents were briefed about the voluntary nature of participation. Participants were assured that the information they share during the interview will be kept confidential and anonymous.

Powered by Editorial Manager® and ProduXion Manager® from Aries Systems Corporation

**Methods section of the manuscript.**<br><br>**Format for specific study types**<br><br>**Human Subject Research (involving human participants and/or tissue)**<br>• Give the name of the institutional review board or ethics committee that approved the study<br>• Include the approval number and/or a statement indicating approval of this research<br>• Indicate the form of consent obtained (written/oral) or the reason that consent was not obtained (e.g. the data were analyzed anonymously)<br><br>**Animal Research (involving vertebrate animals, embryos or tissues)**<br>• Provide the name of the Institutional Animal Care and Use Committee (IACUC) or other relevant ethics board that reviewed the study protocol, and indicate whether they approved this research or granted a formal waiver of ethical approval<br>• Include an approval number if one was obtained<br>• If the study involved *non-human primates*, add *additional details* about animal welfare and steps taken to ameliorate suffering<br>• If anesthesia, euthanasia, or any kind of animal sacrifice is part of the study, include briefly which substances and/or methods were applied<br><br>**Field Research**<br><br>Include the following details if this study involves the collection of plant, animal, or other materials from a natural setting:<br>• Field permit number<br>• Name of the institution or relevant body that granted permission	
**Data Availability**<br><br>Authors are required to make all data underlying the findings described fully available, without restriction, and from the time of publication. PLOS allows rare exceptions to address legal and ethical concerns. See the PLOS Data Policy and FAQ for detailed information.	No - some restrictions will apply

A Data Availability Statement describing where the data can be found is required at submission. Your answers to this question constitute the Data Availability Statement and **will be published in the article**, if accepted.<br><br>**Important:** Stating 'data available on request from the author' is not sufficient. If your data are only available upon request, select 'No' for the first question and explain your exceptional situation in the text box.<br><br>Do the authors confirm that all data underlying the findings described in their manuscript are fully available without restriction?	
**Describe where the data may be found in full sentences. If you are copying our sample text, replace any instances of XXX with the appropriate details.**<br><br>• If the data are **held or will be held in a public repository**, include URLs, accession numbers or DOIs. If this information will only be available after acceptance, indicate this by ticking the box below. For example: *All XXX files are available from the XXX database (accession number(s) XXX, XXX.*).<br>• If the data are all contained **within the manuscript and/or Supporting Information files**, enter the following: *All relevant data are within the manuscript and its Supporting Information files.*<br>• If neither of these applies but you are able to provide **details of access elsewhere**, with or without limitations, please do so. For example:<br><br>*Data cannot be shared publicly because of [XXX]. Data are available from the XXX Institutional Data Access / Ethics Committee (contact via XXX) for researchers who meet the criteria for access to confidential data.*<br><br>*The data underlying the results*	The data were collected by the World Bank. The authors do not have permission to share the data used for the analysis. The data can be obtained on 02/07/2022 after registration at https://microdata.worldbank.org/index.php/catalog/3705

presented in the study are available from (include the name of the third party and contact information or URL).<br>• This text is appropriate if the data are owned by a third party and authors do not have permission to share the data.<br><br>* typeset	
Additional data availability information:	

Manuscript Click here to access/download;Manuscript;3. Manuscript Components_revised_1704.docx

Title

Price elasticity of demand for cigarettes in Nepal: evidence from a lower middle-income country in South Asia using Deaton's demand model

Authors Information

1. **Name:** Vishnu Prasad Sapkota;

   Affiliation: Department of Economics, Tribhuvan University, Nepal Commerce Campus, Min Bhavan Marga, Kathmandu, Nepal.

   Email address: vishnu.sapkota@ncc.tu.edu.np; visapkota@gmail.com

   ORCID ID: https://orcid.org/0000-0002-4014-8267

2. **Name:** Umesh Prasad Bhusal;

   Affiliation: Health Economics Researcher, Kathmandu Nepal;

   Email Address: umbhusal2020@gmail.com;

   ORCID ID: https://orcid.org/0000-0001-9331-6028

3. **Name:** Nirmal Kumar Raut;

   Affiliation: Central Department of Economics; Central Campus, Tribhuvan University, Kirtipur, Kathmandu, Nepal;

   Email address: nirmal.raut@cdec.tu.edu.np;

   ORCID ID: https://orcid.org/0000-0002-4234-4739

Corresponding author

1. **Name:** Vishnu Prasad Sapkota;

   Affiliation: Department of Economics, Tribhuvan University, Nepal Commerce Campus, Min Bhavan Marga, Kathmandu, Nepal.

   Email address: vishnu.sapkota@ncc.tu.edu.np; visapkota@gmail.com

   ORCID ID: https://orcid.org/0000-0002-4014-8267

Abstract

Background: The prevalence of cigarette smoking is declining globally; however, it remains highest in South Asia. Governments in this region rely on taxation as instrument to reduce cigarette demand. The effectiveness of such instruments depends on consumers' underlying preferences and behavioural responses to changes in price – it is commonly expressed in terms of price elasticity of demand. A few studies in this region used widely available cross-sectional surveys to estimate cigarette price elasticity using Deaton's demand model. In this context, we aim to estimate price elasticity of cigarettes in Nepal.

Methods: We applied Deaton's demand model to estimate the cigarette price elasticity using a three-year panel microdata (2016-2018) from Nepal Household Risk and Vulnerability Survey. We used 5653 households observed over three waves; the study units reported purchased quantities of cigarettes and related expenditures. Deaton's demand model exploits spatial variation in unit values of cigarettes to measure price elasticity.

Results: We found that the estimate of price elasticity for cigarette demand was -0.58 with [95% CI: -0.79 – -0.37], and expenditure elasticity estimate was 1.06 [95% CI: 0.54 – 1.57]. These statistics indicate that the cigarette demand is relatively inelastic to price, and unitary elastic to income.

Conclusions: The negative price elasticity estimate for Nepal indicates that the increase in excise taxes can be effective both in reducing cigarette consumption and raising revenues. This

also provides additional evidence that the routine surveys that are designed to monitor living standards can be valuable to estimate price elasticity using the Deaton's method.

Keywords

Price elasticity; Cigarette consumption; Taxation; Tobacco industry; Tobacco policy.

Highlights

What is already known about the topic?

Very few countries in the South Asia have price elasticity estimate for cigarette consumption. Those that report the figure, the responsiveness of cigarette consumption to prices is inelastic in this region, specifically in India and Bangladesh. Such an estimate is not available for Nepal.

What this study adds?

This is the first study that has estimated the responsiveness of price changes to cigarette consumption in Nepal using a panel survey data. The estimated price elasticity was -0.58 with [95% CI: -0.79 – -0.37], and expenditure elasticity estimate was 1.06 [95% CI: 0.54 – 1.57].

How this study might affect research, practice or policy?

The negative price elasticity estimate for Nepal indicates that the increase in excise taxes can be effective both in reducing cigarette consumption and raising tax revenues for governments. This also provides additional evidence that the routine surveys that are designed to monitor living standards can be valuable to estimate price elasticity using the Deaton's method.

1. INTRODUCTION

Cigarette smoking is declining globally; however, its prevalence remains highest in South Asia. Recent evidence report that 27.1% of people aged 15 and above in South Asia smokes cigarettes, with Nepal reporting an even higher rate of 30.4%, compared to the global average of 22.3% (1, 2). Cigarette smoking is documented as one of the important modifiable risk-factors for leading non-communicable diseases(3, 4). Beyond its impact on health, smoking further aggravates living standards and poverty(5-7). These widely reported health and economic impacts, together with the transnational nature of the tobacco industry in the South Asian region, pose multifaced challenges for tobacco control(8). The Framework Convention on Tobacco Control (FCTC) recommends demand reduction of tobacco consumption as an important theme and suggests an array of interventions to make progress in this dimension(9).

Taxation is a widely employed policy instrument to reduce the demand for the tobacco consumption, including cigarette smoking(10). Among the countries in South Asia, taxation policies have the potential to realize dual goals of revenue mobilization and improving public health (by reducing cigarette demand)(11, 12); however, the effectiveness of such policy interventions depends on consumer responses to changes in price. Price elasticity of demand is widely used measure to evaluate such behavioural response. It measures how consumers adjust their purchasing behaviour in response to price changes, and this adjustment is fundamentally rooted in their preferences, including the perceived value, utility, or necessity of the product.

In literature, the price elasticity of demand for cigarette consumption is measured based on time series and cross-sectional data. The latter is commonly used by most low- and middle-income countries employing Deaton's(13) method. This method provides a theory and related procedure to estimate the price elasticity of demand for regular commodities when long-term time series data are challenging to obtain or incomplete. The method provides a robust framework to quantify price responsiveness while uncovering the behavioural factors

underlying purchasing decisions. The method uses rich data from large-scale cross-sectional surveys primarily designed to monitor living standards and consumer prices. By framing price elasticity as an outcome of consumer preferences and price responsiveness, this study not only quantifies the sensitivity of cigarette demand to price changes but also situates these findings within the broader context of consumer decision-making.

The price elasticity of cigarette demand in Nepal holds significant importance and presents a compelling case study in South Asia for at least five reasons. First, cigarette smoking preferences are similar among the adult population in South Asian countries(8), and this is directly reflected in price elasticity estimates. These countries have rich microdata on tobacco consumption, including cigarette smoking; however, studies that estimate the price elasticity of demand for cigarettes in South Asia using Deaton's method are scant. In Nepal, this is the first study to estimate the price elasticity of cigarette demand employing the Deaton's(13) method. In India, earlier estimates show a lower price elasticity of demand (less than -0.20) for cigarettes compared to *bidis* and other smokeless tobacco (SLTs), such as leaf tobacco(14). Subsequent studies show an increase in the price elasticity of demand for cigarettes, ranging from -0.2645 (for the wealthiest group) to -0.832 (for the poorest group)(15, 16). These studies either provide elasticity estimates that are representative of a small subgroup of the population, such as the youth age group, or are outdated by more than a decade. Studies in Bangladesh and Pakistan measure the price elasticity using recent surveys(17, 18). Besides these studies, there appears to be limited evidence of the price elasticity of cigarette consumption from other South Asian countries. Second, cigarette smoking prevalence is higher in this region compared to the global average and also occupies a unique place because of the broad spectrum of cigarettes consumed and the nature of transnational companies engaged in cigarette production in the region (8). Third, the price in PPP $ for the mostly bought cigarette products in Nepal is 7.85, comparable to South Asian countries (8.66 in India, 11.96 in Maldeevs, and 2.91 in

Bandladesh)(19). Similarly, the total tax in Nepal that covers specific excise, ad valorem excise, and value-added tax per cigarette is comparable to South Asian countries(19). In addition, no South Asian countries met the WHO-recommended level of 75% tax share of retail price in 2022(20). Fourth, the cigarette industry in Nepal is an important source of public revenue, occupying significant share of the tobacco industry, and enjoys stronger government support, for example, a slow increase in tax on tobacco products – it is considered reasonable in light of the potential decrease in employment and investment in this sector (8, 21). Fifth, the tax base of tobacco in Nepal, including South Asia, is heavily dependent on tobacco users who smoke cigarettes. Non-cigarette tobacco products account for more than 60% of the tobacco consumption in all countries in this region, yet they contribute less than 20% of the total tobacco tax revenue(8, 22).

In this context, we aim to estimate the cigarette price elasticity in Nepal using Deaton (13) demand model. The approach outlines the related theory and procedure to estimate the price elasticity of demand for regular commodities from cross-sectional surveys; these surveys are often used to monitor poverty and living standards in low-and middle-income countries. In these surveys, households report both expenditure and physical quantities of cigarettes, both of which are affected by the market prices of cigarettes. The two reported variables can be used to derive the unit values by dividing one by the other. However, these unit values cannot be treated as proxies of prices because they are affected by measurement error and quality effects. These survey designs observe clusters of households simultaneously where there is no genuine variation in market prices, but there can be variation in prices between the clusters. The method

exploits this variation to correct the measurement error and quality effects to arrive at the price elasticity of cigarettes. In the next section, we elaborate on the correction method in detail.

2. METHODOLOGY

In this section, we first elaborate on Deaton's approach to measure the price elasticity of demand for regular commodities. Then, we apply the method to estimate the demand equation for cigarette consumption using survey microdata from Nepal. In addition to this, we explain the data source, clusters, and variable definitions.

Specification and estimation

We used the information on reported quantities (q_h) and expenditure (c_h) on cigarettes available in the household survey microdata. These values reflect that households in a given locality face common market prices. However, the information on cigarette prices is unavailable in the dataset. In this context, the proposed method exploits the commonality of prices in a given locality, which is reflected implicitly in q_h and unit values of the cigarettes: $v_h = c_h/q_h$. With this information, Deaton (13) suggested to estimate unit values (v_h) and budget-share (w_h) regression using the following specifications,

$$ln\, v_{hc} = \alpha^1 + \beta^1 ln\, x_{hc} + Z_{hc}\gamma^1 + \psi ln\pi_c + u_{hc}^1 \qquad 1$$

$$w_{hc} = \alpha^o + \beta^o ln\, x_{hc} + Z_{hc}\gamma^o + \theta ln\pi_c + f_c + u_{hc}^o \qquad 2$$

Where, $ln\, v_{hc}$ is the log of unit values derived in the previous step, w_{hc} represents the budget-share for cigarette consumption: $w_{hc} = c_{hc}/x_{hc}$. $ln\, x_{hc}$ is a log of total consumption for the household, and Z_{hc} includes a series of household-specific characteristics such as household size, age and gender composition, household head's characteristics. These variables shift unit values and budget share. In addition to this, the specification also includes consumption

quintiles and year dummies in Z_{hc}. These variables are measured for each household h living in cluster c. The f_c is the cluster-specific fixed-effects, and u^1_{hc} and u^o_{hc} are error terms in the respective equations. These error terms also incorporate the measurement errors in the unit values and budget share. Another important aspect is that equation 1 does not include cluster fixed effects because Deaton (13) elaborates that conditional on prices, unit values depend only on quality effects and measurement errors. $ln\, \pi_c$ appears in two equations, which are unobserved prices and are constant within the clusters because all the households within the cluster face a common price for the cigarette. We estimated the two equations without $ln\, \pi_c$ and, later, recovered the associated coefficients from the expressions proposed by Deaton (13) in their theoretical paper. The relationship between u^1_{hc} and u^o_{hc} was used to correct the price elasticity of demand for cigarettes.

Next, we derived demand for cigarettes and unit values at the cluster level after removing the effects of household consumption and other socio-demographic and household characteristics using equations 1 and 2. These values were then averaged over the clusters because our primary interest was to estimate the elasticity at the cluster-level unit values ($\hat{y}^1_c$) and budget share ($\hat{y}^o_c$) values. These values carry the commonality of price and purchases of cigarettes in the given cluster.

$$\hat{y}^1_c = \frac{1}{n^+_c} \Sigma^{n^+_c}_{h=1} \left(lnv_{hc} - \hat{\beta}^1\, ln\, ln\, x_{hc} - Z_{hc}\hat{\gamma}^1 \right) \qquad 3$$

$$\hat{y}^0_c = \frac{1}{n_c} \Sigma^{n_c}_{h=1} \left(w_{hc} - \hat{\beta}^o\, ln\, ln\, x_{hc} - Z_{hc}\hat{\gamma}^o \right) \qquad 4$$

Based on the cluster-level values derived in the previous step, we estimated a regression coefficient of cluster-level demand ($\hat{y}^o_c$) on cluster level unit values ($\hat{y}^1_c$) based on the expression in equation 5 – it was a standard errors-in-variables regression, where we corrected measurement errors using the covariance and variance of errors.

$$\hat{\phi} = \frac{\left(cov(\hat{y}_c^1, \hat{y}_c^o) - \frac{\hat{\sigma}^{10}}{n_c}\right)}{\left(var(\hat{y}_c^1) - \frac{\hat{\sigma}^{11}}{n_c^+}\right)} \quad 5$$

In the final step, we used a quality correction formula derived by Deaton (13) to obtain the price elasticity estimates of cigarette demand ($\hat{\varepsilon}_p$).

$$\hat{\varepsilon}_p = \left(\frac{\hat{\theta}}{\overline{w}}\right) - \hat{\psi} \quad 6$$

In equation 6, $\overline{w}$is the sample mean of the share of total household consumption expenditure on cigarettes. $\hat{\psi}$ and $\hat{\theta}$ are of the coefficients on the unobserved cluster-level cigarette prices in equations 3.2 and 3.3, respectively. We recovered these values using the following expressions proposed by Deaton (1988).

$$\hat{\psi} = 1 - \frac{\hat{\beta}^1(\underline{w} - \hat{\theta})}{\hat{\beta}^1 + \overline{w}} \quad 7; \qquad \hat{\theta} = \frac{\hat{\phi}}{1 + (\overline{w} - \hat{\phi})\hat{\zeta}} \quad 8; \qquad \hat{\zeta} = \frac{\hat{\beta}^1}{\hat{\beta}^o + \overline{w}(1 - \hat{\beta}^1)} \quad 9$$

We also recovered the expenditure elasticity of demand using the following expression as suggested by Deaton (1988),

$$\widehat{\varepsilon_I} = 1 + \left(\frac{\hat{\beta}^o}{\overline{w}}\right) - \hat{\beta}^1 \quad 10$$

We used R-programming for statistical computing for data management and analysis. In this process, we use the following R packages: *tidyverse* for data management, *fixest* for fixed effect regression models, *survey* to produce survey-weighted estimates, and *boot* to estimate bootstrap standard errors (23-26).

Survey data, cluster definitions, and measurement

We used data from the Nepal Household Risk and Vulnerability Survey – a three-year (2016-2018) household panel. The data was accessed in July, 2022 from The World Bank data

repository[1]. The details about the survey are available in Walker et al. (2019)(27). The survey instrument was based on the World Bank's Living Standards Measurement Study which covered information on socio-demographics, consumption expenditure, income, and labor supply. The sampling frame was all households in rural municipalities, municipalities and sub-metropolitan areas of Nepal. Households were stratified by 11 regions. In the first stage of sampling, 400 wards were randomly selected, with the number within each region predetermined by that region's population share. In the next stage, 15 households were randomly selected within each sampled ward, giving a total of 6,000 sampled households interviewed in 2016 and targeted for reinterview in 2017 and 2018. Attrition was low: 5835 (97.3%) households from the initial sample were reinterviewed in 2017, and 5,696 (94.9%) were reinterviewed in 2018. A total of 5,654 (94.2%) households were interviewed in all three waves. We dropped one household that did not report food expenditure in 2017 and used a balanced, complete-cases sample of 5,653 households, forming 16,959 observations for the analysis.

In this analysis, we use clusters defined by the survey design. This allowed a total of 400 clusters in our analysis. Altogether, in the three waves, there were 42.4 observations in each cluster, which resulted from a follow-up of each household three times in 2016-2018. This approach is in sharp contrast to the approach followed by others Vladisavljevic, Zubović, Đukić and Jovanović in Serbia(28) where clusters are defined as interaction of $clusters \times waves$. Similarly, Chelwa and van Walbeek in Uganda(29) conducted separately analyses for each wave. However, we used repeated observations in the clusters for the following reasons: 1) this increased the cluster sizes, increasing the robustness of the estimates; 2) this increased the reliability of computations of unit values because each household reports the consumption three

[1] https://microdata.worldbank.org/index.php/catalog/3705

times; and 3) this increased the efficiency of the estimates by reducing the error variance. There is the possibility of autocorrelation and year-specific effects in the errors in equations 1 and 2, which we addressed by adjusting the regression equations with year-specific fixed effects and clustering of error terms u_{hc}^{1} and u_{hc}^{o} in equations 1, 2, and 5. This minimized the possibility of bias resulting from the autocorrelation.

The survey questionnaire provides information about the important variables used in the analysis. We used weekly household expenditure on cigarettes, number of cigarettes purchased, and total annual household expenditure – the latter was converted into weekly expenditure to express all the estimates in a common recall period. The monetary values were adjusted to 2017 prices by using the Consumer Price Index available from Nepal Rastra Bank(30). Besides this, as discussed in the equation 1 and 2, we used other socio-demographic variables at the household level – these include household size, age and gender composition of the household members, number of male members, education and gender of household head, maximum education of household members, consumption quintile and place of residence. These variables were recovered from section 1 and section 2 of the survey questionnaire, except for the consumption quintiles, which were based on total annual per capita consumption expenditure. The place of residence of the household was not initially available in the questionnaire – we defined rural households as those living in rural municipalities and urban households as those living in municipalities and sub-metropolitan cities.

3. RESULTS

Table 1 provides the descriptive statistics of the variables used to estimate equations 1 and 2. We found that nearly 17% of households, pooled over three years, reported cigarette consumption in this survey. Regarding expenditure estimates, total household consumption expenditure was 3,562.7 NPR per week, and 15% of households reported non-zero expenditure

on cigarettes. On average, households reported a cigarette consumption of 42.3 pieces per week and spent on average about 170 NPR. Based on this, the unit value of cigarettes per piece was 8.2 NPR, reflecting changes in cigarette prices and choices of cigarette quality purchased on the local market. The share of cigarette spending relative to total household consumption was 5%.

Table 1: Summary statistics of key outcome variables

Variables	Observations	Mean	SD	Q10	Q90
Total consumption per week (NPR)	16959	3562.7	2514.4	1554.9	6056.4
Positive expenses on cigarettes	16959	0.1530	0.3600	0.00	1.00
Cigarette quantities per week (pieces)	2756	42.3	42.0	7.0	120.0
Expenditure on cigarettes per week (NPR)	2756	169.9	342.2	40.00	350.0
Unit value of cigarette (NPR)	2756	8.2	15.9	1.8	10.0
Budget share of cigarette	2756	0.0514	0.0951	0.0118	0.1030
Number of observations in each cluster		42.4	3.1	39	45
Number of households in each cluster		14.1	1.0	13	15
Number of clusters	400				
Number of clusters X years	1200				

Notes: The statistics are pooled over three survey years. Survey weights are used. SD = standard deviation. Q10 and Q90 are quantiles at 10% and 90% points, respectively. NPR = Nepalese Rupee, 2017 prices, NPR1000 = US$9.64.

Table 2 presents the descriptive statistics of the variables used as controls in the regression equation 1 and 2. Again, the statistics were pooled over three surveys. The average household size is about 5, and average number of male members is 2.4. We reported the maximum education of household members irrespective of the member's age – the statistics stand at 7 years. We also reported average percentage of members by age and sex – 0 to 5 years (4% male and 3.5% female), 18 to 49 years (13% male and 25% female), and 50 years or older (11% for

both male and female). Regarding household head characteristics, 21% of household heads were female, and had four years of education on average.

Table 2: Summary statistics of the control variables

Variables	Observations	Mean	SD	Minimum	Maximum
Household demographics					
Household size	16959	4.9711	2.0328	1	22.0000
Number of male members	16959	2.3617	1.2760	0	10.0000
Maximum education of HH members (years)	16959	6.9137	4.5758	0	15.0000
Proportion of household members					
Male [0-5] years	16959	0.0406	0.0894	0	0.6667
Female [0-5] years	16959	0.0364	0.0869	0	0.6667
Male [6-17] years	16959	0.1313	0.1627	0	0.8000
Female [6-17] years	16959	0.1212	0.1585	0	1.0000
Male [18-49] years	16959	0.1840	0.1627	0	1.0000
Female [18-49] years	16959	0.2512	0.1550	0	1.0000
Male [>49] years	16959	0.1185	0.1549	0	1.0000
Female [>49] years	16959	0.1169	0.1659	0	1.0000
Household head's characteristics					
HH head (female) (=1)	16959	0.2115	0.4084	0	1.0000
HH head's education (years)	16959	4.0358	4.3579	0	15.0000
**Notes**: The statistics are pooled over three years. Survey weights are used. SD = standard deviation. HH= household					

Table 3 shows estimated results from the first stage regression for unit value and budget share as per equations 1 and 2. In column (1), we show the estimated coefficients from unit value regression which is a conditional model because it was estimated on the households that report cigarette consumption. In this model, the coefficient for total expenditure is positive and statistically significant; it is a measure of the quality elasticity of expenditure which is about 0.78. Similarly, the coefficient for household size is negative indicating a lower unit value with the increase in household size. However, we do not find statistically significant effects of household socio-demographic composition and household head's characteristics on unit value.

If we ignore the statistical significance, the coefficients are of expected sign; for example, an increase in the percentage of male members has negative effects on the unit values of cigarettes. Similarly, statistically significant and negative coefficients for quintiles indicate that relative to the lowest quintile, households belonging to all other quintiles use cigarettes with lower unit values. In addition to this, concerning survey year, we do not find any statistically significant changes in unit values even though coefficients are relatively large and positive. Finally, cluster fixed effects are jointly statistically significant, confirming pronounced spatial variation in unit values.

In column (2), we present coefficients associated with the budget share regression, which is an unconditional model, as the model was estimated on the whole sample irrespective of whether households reported cigarette consumption. The coefficient on the log of total consumption is positive and statistically significant, indicating that households with higher consumption expenditure tend to allocate more budget for cigarettes – a one percent increase in total consumption increases the budget share by 0.6%. Similarly, the coefficient on household size is negative, although not statistically significant. Besides this, we find statistically significant coefficients for the quintiles – relative to the baseline (lowest quintile), the higher levels of quintiles have negative and statistically significant coefficients indicating that relatively richer households tend to spend less percentage of household total consumption on cigarettes. Similarly, the budget share tends to decrease over time – relative to 2016, the coefficients for 2017 and 2018 are negative and statistically significant. Finally, cluster fixed effects are jointly statistically significant, confirming the spatial variation in budget share.

Table 3: Unit-value and budget share regressions for cigarette consumption in Nepal

**Variables**	**(1)**	**(2)**
	**Unit values of cigarettes in logs**	**Budget share for cigarettes**
Total consumption in logs	0.7785** (0.1709)	0.0066* (0.0021)

Variables	(1) Unit values of cigarettes in logs	(2) Budget share for cigarettes
Household size in logs	-0.5774** (0.1318)	-0.0098 (0.0053)
(Household size in logs)^2	-0.0059 (0.0739)	0.0018 (0.0022)
**Household members' composition (in percent)**		
Males [0-5] years	-0.2599 (0.2319)	-0.0133* (0.0045)
Males [6-17] years	-0.1524 (0.1584)	-0.0047 (0.0070)
Males [18-49] years	-0.1428 (0.0978)	-0.0015 (0.0067)
Males [>49] years	-0.0529 (0.3726)	0.0020 (0.0055)
Females [0-5] years	0.1740 (0.2946)	-0.0043 (0.0089)
Females [6-17] years	-0.0632 (0.1095)	-0.0066 (0.0036)
Females [18-49] years	0.0757 (0.1063)	-0.0051** (0.0010)
**Household head's characteristics**		
HH head (female) (=1)	0.0624 (0.1218)	-0.0035 (0.0017)
HH head's education (years) in logs	0.0229 (0.1260)	0.0005 (0.0003)
(HH head's education (years) in logs)^2	0.0169 (0.0601)	-0.0004* (0.0001)
**Other household characteristics**		
Number of male members	0.0317 (0.0146)	-0.0007 (0.0013)
Maximum education of HH members (years) in logs	-0.0307 (0.1430)	0.0016 (0.0013)
(Maximum education of HH members (years) in logs)^2	-0.0018 (0.0553)	-0.0012 (0.0005)
**Consumption quintiles (First – baseline)**		
Second (=1)	-0.2475** (0.0424)	-0.0025* (0.0008)
Third (=1)	-0.4552* (0.1165)	-0.0048* (0.0015)
Fourth (=1)	-0.5647* (0.1313)	-0.0060 (0.0020)
Fifth (=1)	-0.9139** (0.2115)	-0.0089* (0.0026)
**Place of residence (Rural – baseline)**		
Urban (=1)	-0.0931 (0.1093)	-0.0018 (0.0006)
**Survey year (2016 - baseline)**		
Year - 2017 (=1)	0.1021 (0.0367)	-0.0030*** (0.0001)
Year - 2018 (=1)	0.0800 (0.0871)	-0.0039*** (0.0003)
**Cluster dummies**	**F (377, 2378)**	**F (911, 1844)**
	**2.9958*** **	**1.7631*** **
**R^2 Adjusted**	**0.262**	**0.037**
**Observations**	**2756**	**16959**

Variables	(1)	(2)
	Unit values of cigarettes in logs	Budget share for cigarettes
**Notes.** OLS estimates of coefficients from models 1 and 2. Standard errors (SEs) in parentheses. The autocorrelation of errors over the years is adjusted. The summary of controls is covered in Table 2. The analysis is based on a panel of 5,653 households observed over three years (2016-2018), giving 16,959 observations. Sample and specification in equations 1 and 2. NPR = Nepalese Rupee, 2017 prices, NPR1000 = US$9.64. HH=household. *, **, *** indicate statistical significance at 10%, 5%, and 1%, respectively.		

Table 4 provides summary of the expenditure and price elasticity estimates derived from the procedure described in equations 3 to 10. We use the coefficients together with the key parameters from equation 1 and 2 to find the expenditure elasticity ($\widehat{\varepsilon_I}$)—the estimated value was 1.06, a positive, statistically significant value that means that among the households consuming cigarettes, a 10% higher total household expenditure is associated with an increase in the quantity of cigarettes smoked by nearly the same amount.

Table 4: Elasticity estimates of cigarette demand

	Term	Estimate	SEs	P-values	Confidence interval (95%)	
					Lower	Higher
(1)	Expenditure elasticity ($\widehat{\varepsilon_I}$)	1.0578	(0.2619)	<0.001	0.5445	1.5712
(2)	Price elasticity* ($\widehat{\varepsilon_p}$)	-0.5759	(0.1073)	<0.001	-0.7949	-0.3745

Notes: Row (1) provides expenditure elasticity using equation 10. Row (2) provides the price elasticity of cigarette demand using equations 6-9. Standard errors (SEs) in parentheses. SEs for price elasticity are based on 1,000 bootstrap replications from equations 6-9 and SEs for expenditure elasticity are based on 1,000 bootstrap replications of the equations 1, 2 and 10.

*We also undertake robustness checks of $\widehat{\varepsilon_p}$ across the three survey panels – the estimates range from -0.15 in 2016 to -0.95 in 2018.

After estimating the expenditure elasticity and, together with the estimates from cluster-level averages of budget share and unit values, we estimate the price elasticities as elaborated in equations 3 – 6, and the results are shown below in row (2) of Table 4. The estimated value of price elasticity ($\widehat{\varepsilon_p}$) of cigarettes was -0.58, which indicates that if cigarette prices are increased

by 10%, the corresponding demand will fall only by 5.8%. We computed a 95% confidence interval based on 1,000 bootstrap replications, and the lower and upper intervals are -0.79 and -0.37, respectively.

4. DISCUSSION AND CONCLUSION

We presented the analysis in this paper with the two aims. First, we estimated the price elasticity of demand for cigarettes using microdata from Nepal, where the prevalence and consumption pattern of cigarettes represent the South Asian context. Second, we demonstrated the efficacy of Deaton's method in estimating the price elasticity of cigarettes, by utilizing the cross-sectional data regularly collected for poverty monitoring in Nepal and the other countries in the region. With these aims in mind, we applied Deaton's method to survey data from NHRVS (2016 to 2018) to estimate the price elasticity of demand for cigarettes cigarette in Nepal. We found that the price elasticity ranges from -0.79 to -0.37, with an average value of -0.58. This indicates that the cigarette demand is relatively inelastic in Nepal. This also provides additional evidence that the surveys primarily designed to monitor poverty and living standards can provide useful insights for measuring the price elasticity of cigarettes, as suggested by Deaton(13).

The prevalence of cigarette consumption and expenditure patterns reported from our analyses are comparable to national statistics from the countries in the South Asian region and other LMICs. In our paper, 15 percent of households report non-zero expenses on cigarettes. In the South Asian region, prevalence estimates range from 4.5 percent in Bhutan to 18.7 percent in Maldives, with an average regional prevalence of 10 percent(19). In other LMICs, studies using Deaton's method report a wide variation in non-zero expenses on cigarettes: 1.5 percent in Nigeria, 7 percent in Uganda, and 40 percent in both Bosnia and Herzegovina and Serbia (28, 29, 31-33). The expenditure share on cigarette consumption, however, shows varies to the less

extent: 3.9% in India, 4.8% in Bangladesh, 5.4% in Bosnia and Herzegovina, 3% in Serbia, and 7.6% in Uganda (18, 29, 33, 34). These comparisons indicate that the data and statistics we use are representative of national statistics and hence ensures external validity for the South Asian region.

In the Deaton's method, the efficiency of the estimates relies on two factors: 1) how well the clusters' expenditure share and unit values capture the commonalities in the prices, and 2) the estimates of leading coefficients from unit value and budget share regression. We find that cluster fixed effects strongly determine the variation in unit values and expenditure share, and this ensures that there is a significant commonality in the data at the cluster level that can be exploited to produce the elasticity estimates. Similarly, the coefficients in the budget share and unit values regression align with the available literature in this area. The coefficient on total consumption for unit value regression is 0.78, and that with budget share regression is 0.0066. Studies applying Deaton's method in developing country context report a wide variation in the coefficients for the unit value and budget share regressions: 0.10 and 0.435 in India, 0.147 and 0.0168 in Bangladesh, 0.23 and -0.06 in Uganda, -0.1483 and -0.1453 in Nigeria, 0.06 and 0.004 in Bosnia; 0.11 and 0.002 in Serbia; 0.10 and -0.001 in South Africa, and 0.09 and -0.04 in Croatia (14, 17, 28, 29, 31, 33, 35, 36). These values suggest that our estimates of the coefficients are within the range reported in the literature.

In literature, the estimated value of the price elasticity of demand for cigarettes ranges from -0.165 in Vietnam to -1.37 in Bosnia and Herzegovina(33, 37). These studies used mixed approaches – time series and cross-sectional data. Studies using the time series method report the estimate in the inelastic range (0 to -1) and most of these studies belong to developed countries – the values range from -0.61 to -0.93(38). In developing countries, studies using time series method report a price elasti city of -0.165 in Vietnam and -0.2 in Zambia(39, 40). These figures indicate that consumers are evidently less responsive to the rise in cigarette

prices. On the other hand, the studies using Deaton’s method provide price elasticity estimates in both elastic and inelastic ranges. The majority of the countries report the estimate in an inelastic range – for example, -0.41 and -0.179 in rural and urban India, respectively, -0.89 in urban Bangladesh, -0.33 in Uganda, -0.639 in Serbia, and -0.620 in Nigeria(14, 17, 28, 29, 31). On the other hand, a few studies report elasticities that are unitary elastic and even elastic – for example, -1.06 in Pakistan, -1.38 in rural Bangladesh, -1.366 in Bosnia and Herzegovina(17, 33, 41).

Nepal's estimated value of price elasticity is relevant to the policymakers in the following ways. First, the estimates, together with their lower and upper limits, fall within the range of (-1, 0), suggesting that the cigarette consumption pattern in Nepal is relatively inelastic. This pattern aligns with the those reported in other countries in the South Asian region. This means that consumers reduce cigarette consumption by less than 0.6 percent when the price increases by one percent. Therefore, a tax-increase policy on cigarettes, which raises the market prices, may reduce cigarette consumption to a lesser extent. This suggests that the government should combine the cigarette taxation policy with other measures (such as restrictions on smoking in public places, support for nicotine replacement therapies) to control cigarette consumption. Such a policy response can be expected in other settings where the demand for cigarette consumption is inelastic, such as in India and Bangladesh. Second, policymakers are also interested in the impact of taxation on cigarettes on revenue because it is directly affected by the consumer’s demand response to price changes. Since the demand for cigarettes is relatively inelastic, an increase in excise tax could lead to higher revenue. With inelastic demand, consumers are less likely to reduce their consumption in response to a price increases, meaning that tax revenue could rise despite the higher prices of cigarettes. In Nepal, a recent policy discussion paper on excise tax on cigarettes in the 2024/25 budget examined the implications

of tax increases on government revenue(21) The paper highlights that experience from the last three years demonstrates that increasing the tobacco taxes is an effective way to raise revenues.

Our approach to estimating the price elasticity of demand for cigarettes is not without limitations. First, due to the low frequency of reported cigarette consumption, we used repeated observations from a pool of three-year panel surveys to generate the price elasticity estimates. We corrected for the potential autocorrelation by allowing year-fixed effects into the regression equations. This approach prevents the possible bias. Second, unlike many other studies, we were unable to report disaggregated estimates of the price elasticity by policy relevant variables due to the relatively low frequency of positive cigarette consumption within each cluster. Despite these limitations, our analysis provides a robust estimate of price elasticity that align with the existing literature in the South Asian context and current practices in using price elasticity estimates in the policy context of Nepal.

Acknowledgment: We sincerely thank the World Bank and all the individuals involved in collecting and making the panel data available, which has been invaluable for our research.

Competing Interest: None declared.

Disclaimers. The views expressed in the submitted article are his or her own and not an official position of the institution or funder.

Funding/Support: The authors received no financial support for this research.

References

1. WHO. Tobacco use falling: WHO urges countries to invest in helping more people to quit tobacco2021 2024-12-16. Available from: https://www.who.int/news/item/16-11-2021-tobacco-use-falling-who-urges-countries-to-invest-in-helping-more-people-to-quit-tobacco?utm_source=chatgpt.com.

2. Shariful Islam M, Rashid M, Sizear MI, Hassan R, Rahman M, Parvez SM, et al. Cigarette smoking and associated factors among men in five South Asian countries: A pooled analysis of nationally representative surveys. Plos one. 2022;17(11):e0277758.

3. West R. Tobacco smoking: Health impact, prevalence, correlates and interventions. Psychology & health. 2017;32(8):1018-36.

4. Burki TK. WHO releases latest report on the global tobacco epidemic. The Lancet Oncology. 2021;22(9):1217.

5. Anh PTH, Ross H, Anh NQ, Linh BN, Minh NT. Direct and indirect costs of smoking in Vietnam. Tobacco control. 2016;25(1):96-100.

6. John RM, Sung H-Y, Max WB, Ross H. Counting 15 million more poor in India, thanks to tobacco. Tobacco control. 2011;20(5):349-52.

7. Bobak M, Jha P, Nguyen S, Jarvis M. Poverty and smoking. Oxford University Press; 2000.

8. Gupta PC, Puntambekar N, Assunta M. South Asia's evolving tobacco hydra: moving from quandary to hope. Tobacco Control. 2022;31(2):142-5.

9. WHO. Framework Convention on Tobacco Control. World Health Organization; 2003 2024-12-16.

10. Gravely S, Giovino GA, Craig L, Commar A, D'Espaignet ET, Schotte K, Fong GT. Implementation of key demand-reduction measures of the WHO Framework Convention on

Tobacco Control and change in smoking prevalence in 126 countries: an association study. The Lancet Public Health. 2017;2(4):e166-e74.

11. Rijo M. John HR, Blecher E. A rational taxation system of bidis and cigarettes to reduce smoking deaths in India. Economics for Health. 2011.

12. Jha P, Khan J, Mishra S, Gupta P. Raising taxes key to accelerate tobacco control in South Asia. bmj. 2017;357.

13. Deaton A. Quality, Quantity, and Spatial Variation of Price. The American Economic Review. 1988;78(3):418-30.

14. John RM. Price elasticity estimates for tobacco products in India. Health Policy Plan. 2008;23(3):200-9.

15. Joseph RA, Chaloupka FJ. The influence of prices on youth tobacco use in India. nicotine & tobacco research. 2014;16(Suppl_1):S24-S9.

16. Selvaraj S, Srivastava S, Karan A. Price elasticity of tobacco products among economic classes in India, 2011–2012. BMJ open. 2015;5(12):e008180.

17. Ahmed N, Mozumder TA, Hassan MT, Huque R. Demand for tobacco products in Bangladesh. Tobacco Control. 2022;31(6):737-43.

18. Huque R, Abdullah SM, Hossain MN, Nargis N. Price elasticity of cigarette smoking in Bangladesh: evidence from the Global Adult Tobacco Surveys (GATS). Tob Control. 2024;33(Suppl 2):s51-s8.

19. WHO. Tobacco Retail Price Data 2024 [updated 2024-12-16. Available from: https://apps.who.int/gho/data/view.main.TOBRETAILv.

20. Economics for Health. South Asia Tobacco Control Report. 2022 2024-12-16.

21. National Development Research Institute. Taxing Tobacco in Nepal: What can be done. National Development Research Institute; 2020.

22. National Development Research Institute. Policy Paper: Excise Tax on Cigarettes in the 2024/25 Budget. Kathmandu, Nepal: National Development Research Institute,; 2024.

23. Wickham H, Averick M, Bryan J, Chang W, McGowan LDA, François R, et al. Welcome to the Tidyverse. Journal of open source software. 2019;4(43):1686.

24. Berge L, Krantz S, McDermott G, Lenth R. Package 'fixest'. 2021.

25. Lumley T, Lumley MT. The survey package. hospital. 2007;24:1.

26. Canty A, Ripley B. Package 'boot'. Bootstrap Functions CRAN R Proj. 2017.

27. Walker T, Kawasoe Y, Shrestha J. Risk and vulnerability in Nepal: Findings from the household risk and vulnerability survey: World Bank; 2019.

28. Vladisavljevic M, Zubović J, Đukić M, Jovanović O. Tobacco price elasticity in Serbia: evidence from a middle-income country with high prevalence and low tobacco prices. Tobacco control. 2020;29(Suppl 5):s331-s6.

29. Chelwa G, van Walbeek C. Does cigarette demand respond to price increases in Uganda? Price elasticity estimates using the Uganda National Panel Survey and Deaton's method. BMJ open. 2019;9(3):e026150.

30. NRB. Current Macroeconomic and Financial Situation Tables. Nepal Rastra Bank; 2018 2024-12-16.

31. Adeniji F. Consumption function and price elasticity of tobacco demand in Nigeria. Tobacco Prevention & Cessation. 2019;5.

32. Perera KMN, Guruge GD, Jayawardana PL. Household expenditure on tobacco consumption in a poverty-stricken rural district in Sri Lanka. Asia Pacific Journal of Public Health. 2017;29(2):140-8.

33. Gligorić D, Pepić A, Petković S, Ateljević J, Vukojević B. Price elasticity of demand for cigarettes in Bosnia and Herzegovina: microdata analysis. Tobacco Control. 2020;29(Suppl 5):s304-s9.

34. NSO India. Household Consumption Expenditure Survey, 2022-2023. National Statistical Office, Government of India; 2023.

35. Dare C, Boachie MK, Tingum EN, Abdullah S, van Walbeek C. Estimating the price elasticity of demand for cigarettes in South Africa using the Deaton approach. BMJ open. 2021;11(12):e046279.

36. Barać ŽA, Burnać P, Rogošić A, Šodan S, Vuko T. Cigarette price elasticity in Croatia–analysis of household budget surveys. Journal of applied economics. 2021;24(1):318-28.

37. Viet Nguyen C, Le TT, Nguyen NH. The impact of cigarette prices on smoking participation and tobacco expenditure in Vietnam. Plos one. 2021;16(12):e0260415.

38. Kohler A, Vinci L, Mattli R. Cross-country and panel data estimates of the price elasticity of demand for cigarettes in Europe. BMJ open. 2023;13(6):e069970.

39. Nguyen N, Nguyen AN. Price elasticity estimates of cigarette demand in Vietnam using survey data. Tobacco Induced Diseases. 2021;19(1).

40. Stoklosa M, Goma F, Nargis N, Drope J, Chelwa G, Chisha Z, Fong GT. Price, tax and tobacco product substitution in Zambia: findings from the ITC Zambia Surveys. Tobacco Control. 2019;28(Suppl 1):s45-s52.

41. Nayab D, Nasir M, Memon JA, Khalid M, Hussain A. Estimating the price elasticity for cigarette and chewed tobacco in Pakistan: evidence from microlevel data. Tob Control. 2020;29(Suppl 5):s319-s25.

6. Supplementary Material or Appendices

Not Available

Manuscript Click here to access/download;Manuscript;3. Manuscript Components_revised_1010.docx

Title

Price elasticity of demand for cigarettes in Nepal: evidence from a lower middle-income country in South Asia using Deaton's demand model

Authors Information

1. **Name:** Vishnu Prasad Sapkota;

   Affiliation: Department of Economics, Tribhuvan University, Nepal Commerce Campus, Min Bhavan Marga, Kathmandu, Nepal.

   Email address: vishnu.sapkota@ncc.tu.edu.np; visapkota@gmail.com

   ORCID ID: https://orcid.org/0000-0002-4014-8267

2. **Name:** Umesh Prasad Bhusal;

   Affiliation: Health Economics Researcher, Kathmandu Nepal.

   Email Address: umbhusal2020@gmail.com;

   ORCID ID: https://orcid.org/0000-0001-9331-6028

3. **Name:** Nirmal Kumar Raut;

   Affiliation: Central Department of Economics; Central Campus, Tribhuvan University, Kirtipur, Kathmandu, Nepal.

   Email address: nirmal.raut@cdec.tu.edu.np;

   ORCID ID: https://orcid.org/0000-0002-4234-4739

Corresponding author

1. **Name:** Vishnu Prasad Sapkota;

   Affiliation: Department of Economics, Tribhuvan University, Nepal Commerce Campus, Min Bhavan Marga, Kathmandu, Nepal.

   Email address: vishnu.sapkota@ncc.tu.edu.np; visapkota@gmail.com

   ORCID ID: https://orcid.org/0000-0002-4014-8267

Abstract

Background: The prevalence of cigarette smoking is declining globally; however, it remains highest in South Asia. Governments in this region rely on taxation as an instrument to reduce cigarette demand. The effectiveness of such instruments depends on consumers' underlying preferences and behavioural responses to changes in price – it is commonly expressed in terms of price elasticity of demand. A few studies in this region used available cross-sectional and panel surveys to estimate cigarette price elasticity using Deaton's demand model. In this context, we aim to estimate the price elasticity of cigarettes in Nepal.

Methods: We applied Deaton's demand model to estimate the cigarette price elasticity using a three-year panel microdata (2016-2018) from the Nepal Household Risk and Vulnerability Survey. We used data from 5,653 households observed over three waves; the study units reported the purchased quantities of cigarettes and related expenditures. Deaton's demand model exploits spatial variation in unit values of cigarettes to measure price elasticity. We adjusted the basic model for possible bias that may arise from panel nature of the data.

Results: We found that the estimate of price elasticity for cigarette demand was -0.58 with [95% CI: -0.79 -0.37], and the expenditure elasticity estimate was 1.06 [95% CI: 0.54 – 1.57]. These statistics indicate that the cigarette demand is relatively inelastic to price, and unitary elastic to income.

Conclusions: The negative price elasticity estimate for Nepal indicates that the increase in excise taxes can be effective both in reducing cigarette consumption and raising revenues. This also provides additional evidence that the routine surveys, including panel data, that are

designed to monitor living standards can be valuable to estimate price elasticity using the Deaton’s method.

Keywords

Price elasticity; Cigarette consumption; Taxation; Tobacco industry; Tobacco policy.

Highlights

What is already known about the topic?

Very few countries in South Asia have a price elasticity estimate for cigarette consumption. Those who report the figure, the responsiveness of cigarette consumption to prices is inelastic in this region, specifically in India and Bangladesh. Such an estimate is not available for Nepal.

What this study adds?

This is the first study to estimate the responsiveness of price changes to cigarette consumption in Nepal using panel survey data. The estimated price elasticity was -0.58 with [95% CI: -0.79 -0.37], and the expenditure elasticity estimate was 1.06 [95% CI: 0.54 – 1.57].

How this study might affect research, practice or policy?

The negative price elasticity estimate for Nepal suggests that increasing excise taxes can be effective in reducing cigarette consumption and increasing tax revenues for governments. This also provides additional evidence that the panel surveys that are designed to monitor living standards can be valuable to estimate price elasticity using the Deaton’s method.

1. INTRODUCTION

Cigarette smoking is declining globally; however, its prevalence remains highest in South Asia. Recent evidence reports that 27.1% of people aged 15 and above in South Asia smoke cigarettes, with Nepal reporting an even higher rate of 30.4%, compared to the global average of 22.3% (1, 2). Cigarette smoking is a major modifiable risk-factors for leading non-communicable diseases (3, 4). Beyond its impact on health, smoking worsens living standards and poverty (5-7). These health and economic impacts, together with the transnational nature of the tobacco industry in the South Asian region, pose multifaced challenges for tobacco control (8). The Framework Convention on Tobacco Control (FCTC) recommends demand reduction of tobacco consumption as an important theme and suggests interventions such as taxation, health warnings, and advertising bans, to advance this agenda (9).

Taxation is a widely employed policy instrument to reduce tobacco consumption, including cigarette smoking (10). In South Asia, it can achieve dual goals of revenue mobilization and improved public health by reducing cigarette demand (11, 12). However, its effectiveness depends on consumer responses to price changes. Price elasticity of demand, a common measure of these responses, shows how consumers adjust their purchasing behaviour to price changes, reflecting preferences, perceived value, utility, or necessity of the product.

In the literature, the price elasticity of demand for cigarette consumption is measured based on time series and cross-sectional data. The latter is commonly used by most low- and middle-income countries employing Deaton's method (13). This method provides a framework to estimate the price elasticity of demand for regular commodities when long-term time series data are unavailable or incomplete. It provides a robust framework to quantify price responsiveness while uncovering the behavioural factors underlying purchasing decisions. It uses rich data from large-scale cross-sectional surveys, primarily designed to monitor living standards and consumer prices. By framing price elasticity as an outcome of consumer

preferences and price responsiveness, this study not only quantifies the sensitivity of cigarette demand to price changes but also situates these findings within the broader context of consumer decision-making.

The price elasticity of cigarette demand in Nepal holds significant importance and presents a compelling case study in South Asia for the following reasons. First, cigarette smoking preferences are similar among adults in South Asian countries (8), yet studies using Deaton's method remain scant despite the availability of rich microdata on tobacco consumption. This is the first study in Nepal to estimate the price elasticity of cigarette demand employing the Deaton's method (13). Regional evidence is limited and mixed: in India, earlier estimates showed a lower price elasticity of demand for cigarettes (-0.179) compared to *bidis* and leaf tobacco (-0.87), especially in urban India (14), while subsequent studies showed an increase in the price elasticity of demand for cigarettes, ranging from -0.2645 (for the wealthiest group) to -0.832 (for the poorest group) (15, 16), though most are outdated or limited to subgroups such as youth. Studies in Bangladesh and Pakistan rely on recent surveys (17, 18), but evidence from other South Asian countries remains scarce. Second, cigarette smoking prevalence in South Asia is higher compared to the global average, with diverse products and strong transnational industry presence (8). Third, cigarette prices in purchasing power parity (PPP) dollar are broadly comparable across the region (7.85 in Nepal, 8.66 in India, 11.96 in Maldives, and 2.91 in Bangladesh) (19), and tax rates are similar, yet no country met the WHO-recommended 75% share of the retail price in 2022 (20).

Table 1 shows the trend of cigarette prices and taxes in Nepal over the period of 10 years. The cigarette prices have been increasing at constant rate of ~1.00 USD (in PPP) each year between 2012 to 2022. This means that the household face a reasonably constant increment in prices before and during the survey period. During the same period, the excise taxes have remained

stable between 26 – 30 percent of cigarette prices. These statistics point to the fact that price changes and tax on cigarette were relatively stable during the study period.

Table 1: Cigarette price and tax in Nepal between 2012 and 2022.

**Year**	**2012**	**2014**	**2016**	**2018**	**2020**	**2022**
Retail price ~~of cigarette~~ (per 20 sticks in PPP dollars)	3.65	4.69	5.63	6.78	8.38	10.43
Taxes as percentage of cigarette price (%)	29.86	27.79	26	30	27	31.39
Source (21): https://www.who.int/data/gho/data/themes/topics/raise-taxes-on-tobacco						

Fourth, the cigarette industry in Nepal contributes significantly to public revenue and receives government support, partly due to concerns over employment and investment (8, 22). Fifth, although non-cigarette tobacco products account for more than 60% of the tobacco consumption, , they contribute less than 20% of the total tobacco tax revenue (8, 23).

In this context, we aim to estimate the price elasticity of cigarette demand in Nepal using the demand model of Deaton (13). The approach outlines the theory and procedure to estimate the price elasticity of demand for regular commodities from cross-sectional surveys, which are often used to monitor poverty and living standards in low-and middle-income countries. In these surveys, households report both expenditure and physical quantities of cigarettes, which are affected by the market prices. The two reported variables can be used to derive the unit values by dividing expenditure by quantity. However, these unit values cannot be treated as proxies for prices because they are affected by measurement error and quality effects. These surveys observe clusters of households simultaneously, where there is no genuine variation in market prices, but variation can exist between clusters. The Deaton method exploits this

variation to correct the measurement error and quality effects to arrive at the price elasticity of cigarettes. In the next section, we elaborate on the correction method in detail.

2. METHODOLOGY

In this section, we first elaborate on Deaton's approach to measure the price elasticity of demand for regular commodities. Then, we apply the method to estimate the demand equation for cigarette consumption using panel survey from Nepal. In addition to this, we also elaborate additional assumptions and the adjustments we made in the original model that was applied to cross-sectional surveys. Besides this, we explain the data source, clusters, and variable definitions.

Specification and estimation

We used the information on reported quantities (q_{ht}) and expenditure (c_{ht}) on cigarettes available in the household survey over the three waves. These values reflect that households in each locality face common market prices in each wave. However, the information on cigarette prices is not available in the dataset. In this context, the proposed method exploits the commonality of prices in each locality, which is reflected implicitly in q_{ht} and unit values of the cigarettes ($v_{ht} = c_{ht}/q_{ht}$). In Deaton's model, the key identifying assumption is that inter-cluster variation in prices serves as an instrumental variable (IV) for demand. This assumption remains intact in our panel context, where households face the same cluster price at each time point, but with possible temporal variation. Such variation requires careful treatment in unit values and budget share regressions (equation 1 and 2) but does not invalidate the basic identification and estimation strategy, which we discuss below. With this information and underlying assumptions, we estimated unit values (v_{ht}) and budget-share (w_{ht}) regression using the following specifications,

$$ln\, v_{hct} \;=\; \alpha^1 + \beta^1 ln\, x_{hct} \; + Z_{hct}\gamma^1 + \psi ln\pi_c + y_t + u^1_{hct} \qquad 1$$

$$w_{hct} = \; \alpha^o + \beta^o ln\, x_{hct} \; + Z_{hct}\gamma^o + \theta ln\pi_c + f_c + y_t + u^o_{hct} \qquad 2$$

Where, $ln\ v_{hct}$ is the log of unit values derived in the previous step, w_{hct} represents the budget-share for cigarette consumption: $w_{hct} = c_{hct}/x_{hct}$. $ln\ x_{hct}$ is a log of total consumption for the household, and Z_{hct} includes a series of household-specific characteristics such as household size, age and gender composition, household head's characteristics. These variables shift unit values and budget share. The f_c is the cluster-specific fixed-effects, y_t is year specific fixed effect, and u^1_{hct} and u^o_{hct} are error terms in the respective equations. Deaton in his original set-up assumed that these error terms also incorporate the measurement errors in the unit values and budget share. Another important aspect is that equation 1 does not include cluster fixed effects because Deaton (13) elaborates that conditional on cross-sectional and temporal variation in prices, unit values depend only on quality effects and measurement errors. $ln\ \pi_c$ appears in two equations, which are unobserved prices and are constant within the clusters because all the households within the cluster face a common price for the cigarette after removing year specific fixed effects. We estimated the two equations without $ln\ \pi_c$ and, later, recovered the associated coefficients from the expressions proposed by Deaton (13) in their theoretical paper. The relationship between u^1_{hct} and u^o_{hct} was used to correct the price elasticity of demand for cigarettes.

In the pooled OLS set-up of equations 1 and 2, we adjust for possible bias that may arise from repeated measurements over the three waves, possibly due to temporal trends in unit values and budget shares, temporal correlation in the error terms of the respective equations, and

unobserved changes in cigarette consumption patterns which may be correlated with the covariates. We correct for these as follows:

1. We add year-specific fixed effects to ensure that between-cluster variation in the unit value and budget share regressions is free from temporal trends. This ensures that the basic requirement to apply the Deaton demand model is maintained.

2. In equation 1 and 2, within-cluster variation may be due to (i) variation in cigarette consumption preferences over the three-year period, (ii) temporal correlation in the error terms in equations 1 and 2, and (iii) measurement error (correlation between the error terms of equations 1 and 2). Of these, (iii) is key to Deaton's correction strategy for the *measurement error*. We minimize bias that may result from (i) and (ii) with the following adjustments and assumptions:

   - We adjust for temporal correlation in u^1_{hct} and u^o_{hct} by allowing the errors to cluster over time while estimating the *measurement error*.
   - We assume that policy-driven shifts in cigarette preferences are minimal during the survey period – a stable, marginal change in cigarette prices and taxes between 2016–2018 (shown in Table 1) provides evidence that this assumption is met.
   - Cigarette consumption behaviour exhibits a stable pattern over short time interval. A one-year gap between each survey wave makes preference changes unlikely –

longitudinal studies show that transitions between smoker and non-smoker states are rare within one year (24).

Together, with these assumptions and adjustments, the coefficient estimates from the pooled OLS are unbiased and measurement error corrections still maintain the properties of the original Deaton (1988) model.

Next, we derived demand for cigarettes and unit values at the cluster level after removing the effects of household consumption, socio-demographic and household head's characteristics and year specific fixed effects using equations 1 and 2. These values were then averaged over the clusters because our primary interest was to estimate the elasticity at the cluster-level unit values ($\hat{y}_c^1$) and budget share ($\hat{y}_c^o$) values. These values carry the commonality of price and purchases of cigarettes in the given cluster. From equations (3) and (4) onwards, the analysis relies on cluster-level averages of unit values and budget shares, after purging household characteristics and year effects. At this stage, our procedure is identical to Deaton's cross-sectional approach.

$$\hat{y}_c^1 = \frac{1}{n_c^+} \Sigma_{h=1}^{n_c^+} \left(lnv_{hc} - \hat{\beta}^1 \, ln \, ln \, x_{hc} \; - \; Z_{hc} \hat{\gamma}^1 \right) \qquad 3$$

$$\hat{y}_c^0 = \frac{1}{n_c} \Sigma_{h=1}^{n_c} \left(w_{hc} - \hat{\beta}^o \, ln \, ln \, x_{hc} \; - \; Z_{hc} \hat{\gamma}^o \right) \qquad 4$$

Based on the cluster-level values derived in the previous step, we estimated a regression coefficient of cluster-level demand ($\hat{y}_c^o$) on cluster level unit values ($\hat{y}_c^1$) based on the expression in equation 5 – it was a standard errors-in-variables regression, where we corrected measurement errors using the covariance and variance of errors. In this step, we allowed the households to be correlated across the time to correct the autocorrelation in error terms in equation 1 and 2.

$$\hat{\phi} = \frac{\left(cov(\hat{y}_c^1, \hat{y}_c^o) - \frac{\hat{\sigma}^{10}}{n_c}\right)}{\left(var(\hat{y}_c^1) - \frac{\hat{\sigma}^{11}}{n_c^+}\right)} \quad 5$$

In the final step, we used a quality correction formula derived by Deaton (13) to obtain the price elasticity estimates of cigarette demand ($\hat{\varepsilon}_p$).

$$\hat{\varepsilon}_p = \left(\frac{\hat{\theta}}{\overline{w}}\right) - \hat{\psi} \quad 6$$

In equation 6, $\overline{w}$is the sample mean of the share of total household consumption expenditure on cigarettes. $\hat{\psi}$ and $\hat{\theta}$ are of the coefficients on the unobserved cluster-level cigarette prices in equations 1 and 2, respectively. We recovered these values using the following expressions proposed by Deaton (1988).

$$\hat{\psi} = 1 - \frac{\hat{\beta}^1(\overline{w} - \hat{\theta})}{\hat{\beta}^1 + \overline{w}} \quad 7; \qquad \hat{\theta} = \frac{\hat{\phi}}{1 + (\overline{w} - \hat{\phi})\hat{\zeta}} \quad 8; \qquad \hat{\zeta} = \frac{\hat{\beta}^1}{\hat{\beta}^o + \overline{w}(1 - \hat{\beta}^1)} \quad 9$$

We also recovered the expenditure elasticity of demand using the following expression as suggested by Deaton (1988),

$$\hat{\varepsilon_I} = 1 + \left(\frac{\hat{\beta}^o}{\overline{w}}\right) - \hat{\beta}^1 \quad 10$$

We used R-programming for statistical computing for data management and analysis. In this process, we use the following R packages: *tidyverse* for data management, *fixest* for fixed effect regression models, *survey* to produce survey-weighted estimates, and *boot* to estimate bootstrap standard errors (24-27).

Survey data, cluster definitions, and measurement

We used data from the Nepal Household Risk and Vulnerability Survey – a three-year (2016-2018) household panel. The data was accessed in July, 2022 from The World Bank data

repository[1]. The details about the survey are available in Walker et al. (2019) (28). The survey instrument was based on the World Bank's Living Standards Measurement Study which covered information on socio-demographics, consumption expenditure, income, and labor supply. The sampling frame was all households in rural municipalities, municipalities and sub-metropolitan areas of Nepal. Households were stratified by 11 regions. In the first stage of sampling, 400 wards were randomly selected, with the number within each region predetermined by that region's population share. In the next stage, 15 households were randomly selected within each sampled ward, giving a total of 6,000 sampled households interviewed in 2016 and targeted for reinterview in 2017 and 2018. Attrition was low: 5835 (97.3%) households from the initial sample were reinterviewed in 2017, and 5,696 (94.9%) were reinterviewed in 2018. A total of 5,654 (94.2%) households were interviewed in all three waves. We dropped one household that did not report food expenditure in 2017 and used a balanced, complete-cases sample of 5,653 households, forming 16,959 observations for the analysis.

In this analysis, we use clusters defined by the survey design. This allowed a total of 400 clusters in our analysis. Altogether, in the three waves, there were 42.4 observations in each cluster, which resulted from a follow-up of each household three times in 2016-2018. This approach is in sharp contrast to the approach followed by others Vladisavljevic, Zubović, Đukić and Jovanović in Serbia (29) where clusters are defined as interaction of $clusters \times waves$. Similarly, Chelwa and van Walbeek in Uganda (30) conducted separately analyses for each wave. However, we used repeated observations in the clusters for the following reasons: 1) this increased the cluster sizes, increasing the robustness of the estimates; 2) this increased the reliability of computations of unit values because each household reports the consumption three

[1] https://microdata.worldbank.org/index.php/catalog/3705

times; and 3) this increased the efficiency of the estimates by reducing the error variance. There is the possibility of autocorrelation and year-specific effects in the errors in equations 1 and 2, which we addressed by adjusting the regression equations with year-specific fixed effects and clustering of error terms u_{hc}^{1} and u_{hc}^{o} in equations 1, 2, and 5. This minimized the possibility of bias resulting from the autocorrelation.

The survey questionnaire provides information about the important variables used in the analysis. We used weekly household expenditure on cigarettes, number of cigarettes purchased, and total annual household expenditure – the latter was converted into weekly expenditure to express all the estimates in a common recall period. The monetary values were adjusted to 2017 prices by using the Consumer Price Index available from Nepal Rastra Bank (31). Besides this, as discussed in the equation 1 and 2, we used other socio-demographic variables at the household level – these include household size, age and gender composition of the household members, number of male members, education and gender of household head, maximum education of household members, consumption quintile and place of residence. These variables were recovered from section 1 and section 2 of the survey questionnaire, except for the consumption quintiles, which were based on total annual per capita consumption expenditure. The place of residence of the household was not initially available in the questionnaire – we defined rural households as those living in rural municipalities and urban households as those living in municipalities and sub-metropolitan cities.

3. RESULTS

Table 2 provides the descriptive statistics of the variables used to estimate equations 1 and 2. We found that nearly 17% of households, pooled over three years, reported cigarette consumption in this survey. Regarding expenditure estimates, total household consumption expenditure was 3,562.7 NPR per week, and 15% of households reported non-zero expenditure

on cigarettes. On average, households reported a cigarette consumption of 42.3 pieces per week and spent on average about 170 NPR. Based on this, the unit value of cigarettes per piece was 8.2 NPR, reflecting changes in cigarette prices and choices of cigarette quality purchased on the local market. The share of cigarette spending relative to total household consumption was 5%.

Table 2: Summary statistics of key outcome variables

Variables	Observations	Mean	SD	Q10	Q90
Total consumption per week (NPR)	16959	3562.7	2514.4	1554.9	6056.4
Positive expenses on cigarette	16959	0.1530	0.3600	0.00	1.00
Cigarette quantities per week (pieces)	2756	42.3	42.0	7.0	120.0
Expenditure on cigarettes per week (NPR)	2756	169.9	342.2	40.00	350.0
Unit value of cigarette (NPR)	2756	8.2	15.9	1.8	10.0
Budget share of cigarette	2756	0.0514	0.0951	0.0118	0.1030
Number of observations in each cluster		42.4	3.1	39	45
Number of households in each cluster		14.1	1.0	13	15
Number of clusters	400				
Number of clusters X years	1200				

Note: The statistics are pooled over three survey years. Survey weights are used. SD = standard deviation. Q10 and Q90 are quantiles at 10% and 90% points, respectively. NPR = Nepalese Rupee, 2017 prices, NPR1000 = US$9.64.

Table 3 presents the descriptive statistics of the variables used as controls in the regression equation 1 and 2. Again, the statistics were pooled over three surveys. The average household size is about 5, and average number of male members is 2.4. We reported the maximum education of household members irrespective of the member's age – the statistics stand at 7 years. We also reported average percentage of members by age and sex – 0 to 5 years (4% male and 3.5% female), 18 to 49 years (13% male and 25% female), and 50 years or older (11% for

both male and female). Regarding household head characteristics, 21% of household heads were female, and had four years of education on average.

Table 3: Summary statistics of the control variables

Variables	Observations	Mean	SD	Minimum	Maximum
Household demographics					
Household size	16959	4.9711	2.0328	1	22.0000
Number of male members	16959	2.3617	1.2760	0	10.0000
Maximum education of HH members (years)	16959	6.9137	4.5758	0	15.0000
Proportion of household members					
Male [0-5] years	16959	0.0406	0.0894	0	0.6667
Female [0-5] years	16959	0.0364	0.0869	0	0.6667
Male [6-17] years	16959	0.1313	0.1627	0	0.8000
Female [6-17] years	16959	0.1212	0.1585	0	1.0000
Male [18-49] years	16959	0.1840	0.1627	0	1.0000
Female [18-49] years	16959	0.2512	0.1550	0	1.0000
Male [>49] years	16959	0.1185	0.1549	0	1.0000
Female [>49] years	16959	0.1169	0.1659	0	1.0000
Household head's characteristics					
HH head (female) (=1)	16959	0.2115	0.4084	0	1.0000
HH head's education (years)	16959	4.0358	4.3579	0	15.0000
**Note**: The statistics are pooled over three years. Survey weights are used. SD = standard deviation. HH= household					

Table 4 presents the estimated results from the first-stage regression for unit value and budget share, as per Equations 1 and 2. In column (1), we show the estimated coefficients from unit value regression, which is a conditional model because it was estimated on the households that report cigarette consumption. In this model, the coefficient for total expenditure is positive and statistically significant; it is a measure of the quality elasticity of expenditure, which is about 0.78. Similarly, the coefficient for household size is negative, indicating a lower unit value with the increase in household size. However, we do not find statistically significant effects of household socio-demographic composition and the household head's characteristics on unit

value. If we ignore the statistical significance, the coefficients are of the expected sign; for example, an increase in the percentage of male members has a negative effect on the unit values of cigarettes. Similarly, statistically significant and negative coefficients for quintiles indicate that relative to the lowest quintile, households belonging to all other quintiles use cigarettes with lower unit values. In addition to this, concerning the survey year, we do not find any statistically significant changes in unit values, even though the coefficients are relatively large and positive. Finally, cluster fixed effects are jointly statistically significant, confirming pronounced spatial variation in unit values.

In column (2), we present coefficients associated with the budget share regression, which is an unconditional model, as the model was estimated on the whole sample irrespective of whether households reported cigarette consumption. The coefficient on the log of total consumption is positive and statistically significant, indicating that households with higher consumption expenditure tend to allocate more budget for cigarettes – a one percent increase in total consumption increases the budget share by 0.6%. Similarly, the coefficient on household size is negative, although not statistically significant. Besides this, we find statistically significant coefficients for the quintiles – relative to the baseline (lowest quintile), the higher levels of quintiles have negative and statistically significant coefficients, indicating that relatively richer households tend to spend a smaller percentage of their household total consumption on cigarettes. Similarly, the budget share tends to decrease over time – relative to 2016, the coefficients for 2017 and 2018 are negative and statistically significant. Finally, cluster fixed effects are jointly statistically significant, confirming the spatial variation in budget share.

Table 4: Unit-value and budget share regressions for cigarette consumption in Nepal

**Variables**	**(1)**	**(2)**
	**Unit values of cigarettes in logs**	**Budget share for cigarettes**
Total consumption in logs	0.7785** (0.1709)	0.0066* (0.0021)

Variables	(1) Unit values of cigarettes in logs	(2) Budget share for cigarettes
Household size in logs	-0.5774** (0.1318)	-0.0098 (0.0053)
(Household size in logs)^2	-0.0059 (0.0739)	0.0018 (0.0022)
**Household members' composition (in percent)**		
Males [0-5] years	-0.2599 (0.2319)	-0.0133* (0.0045)
Males [6-17] years	-0.1524 (0.1584)	-0.0047 (0.0070)
Males [18-49] years	-0.1428 (0.0978)	-0.0015 (0.0067)
Males [>49] years	-0.0529 (0.3726)	0.0020 (0.0055)
Females [0-5] years	0.1740 (0.2946)	-0.0043 (0.0089)
Females [6-17] years	-0.0632 (0.1095)	-0.0066 (0.0036)
Females [18-49] years	0.0757 (0.1063)	-0.0051** (0.0010)
**Household head's characteristics**		
HH head (female) (=1)	0.0624 (0.1218)	-0.0035 (0.0017)
HH head's education (years) in logs	0.0229 (0.1260)	0.0005 (0.0003)
(HH head's education (years) in logs)^2	0.0169 (0.0601)	-0.0004* (0.0001)
**Other household characteristics**		
Number of male members	0.0317 (0.0146)	-0.0007 (0.0013)
Maximum education of HH members (years) in logs	-0.0307 (0.1430)	0.0016 (0.0013)
(Maximum education of HH members (years) in logs)^2	-0.0018 (0.0553)	-0.0012 (0.0005)
**Consumption quintiles (First – baseline)**		
Second (=1)	-0.2475** (0.0424)	-0.0025* (0.0008)
Third (=1)	-0.4552* (0.1165)	-0.0048* (0.0015)
Fourth (=1)	-0.5647* (0.1313)	-0.0060 (0.0020)
Fifth (=1)	-0.9139** (0.2115)	-0.0089* (0.0026)
**Place of residence (Rural – baseline)**		
Urban (=1)	-0.0931 (0.1093)	-0.0018 (0.0006)
**Survey year (2016 - baseline)**		
Year - 2017 (=1)	0.1021 (0.0367)	-0.0030*** (0.0001)
Year - 2018 (=1)	0.0800 (0.0871)	-0.0039*** (0.0003)
**Cluster dummies**	**F (377, 2378)**	**F (911, 1844)**
	**2.9958*** **	**1.7631*** **
**R^2 Adjusted**	**0.262**	**0.037**
**Observations**	**2756**	**16959**

Variables	(1)	(2)
	**Unit values of cigarettes in logs**	**Budget share for cigarettes**

Note: OLS estimates of coefficients from models 1 and 2. Standard errors (SEs) in parentheses. The autocorrelation of errors over the years is adjusted. The summary of controls is covered in Table 2. The analysis is based on a panel of 5,653 households observed over three years (2016-2018), giving 16,959 observations. Sample and specification in equations 1 and 2. NPR = Nepalese Rupee, 2017 prices, NPR1000 = US$9.64. HH=household. *, **, *** indicate statistical significance at 10%, 5%, and 1%, respectively.

Table 5 provides summary of the expenditure and price elasticity estimates derived from the procedure described in equations 3 to 10. We use the coefficients together with the key parameters from equations 1 and 2 to find the expenditure elasticity ($\widehat{\varepsilon_I}$)—the estimated value was 1.06, a positive, statistically significant value that means that among the households consuming cigarettes, a 10% higher total household expenditure is associated with an increase in the quantity of cigarettes smoked by nearly the same amount.

Table 5: Elasticity estimates of cigarette demand

	Term	Estimate	SEs	P-values	Confidence interval (95%)	
					Lower	Higher
(1)	Expenditure elasticity ($\widehat{\varepsilon_I}$)	1.0578	(0.2619)	<0.001	0.5445	1.5712
(2)	Price elasticity* ($\widehat{\varepsilon_p}$)	-0.5759	(0.1073)	<0.001	-0.7949	-0.3745

Note: Row (1) provides expenditure elasticity using equation 10. Row (2) provides the price elasticity of cigarette demand using equations 6-9. Standard errors (SEs) in parentheses. SEs for price elasticity are based on 1,000 bootstrap replications from equations 6-9 and SEs for expenditure elasticity are based on 1,000 bootstrap replications of the equations 1, 2 and 10.

*We also undertake robustness checks of $\widehat{\varepsilon_p}$ across the three survey panels – the estimates range from -0.15 in 2016 to -0.95 in 2018.

After estimating the expenditure elasticity and, together with the estimates from cluster-level averages of budget share and unit values, we estimate the price elasticities as elaborated in equations 3 – 6, and the results are shown below in row (2) of Table 5. The estimated value of price elasticity ($\widehat{\varepsilon_p}$) of cigarettes was -0.58, which indicates that if cigarette prices are increased by 10%, the corresponding demand will fall only by 5.8%. We computed a 95% confidence

interval based on 1,000 bootstrap replications, and the lower and upper intervals are -0.79 and -0.37, respectively.

4. DISCUSSION AND CONCLUSION

In this paper, our objective is to estimate the price elasticity of demand for cigarettes using panel survey microdata from Nepal. We found that the demand is inelastic as price elasticity ranges from -0.79 to -0.37, with an average value of -0.58.

The dataset we used for the analysis ensures that findings are comparable to the south Asian region. We compare the statistics reported by other related papers in south Asian and international context. The prevalence of cigarette consumption and expenditure patterns reported from our analyses are comparable to national statistics from the countries in the South Asian region and other LMICs (18, 19, 29, 30, 32-35).The findings related to prevalence and expenditure patterns using data from Nepal exhibit consistency when compared with its South Asian Counterparts as well as other LMICs. Nepalese households incur non-zero spending are 15 percent, which is higher than the regional average of 10 percent, the statistics lie within the range from 4.5 percent in Bhutan to 18.7 percent in the Maldives (19). The prevalence reported elsewhere exhibits wide variation, ranging from 1.5 percent in Nigeria to 7 percent in Uganda and 40 percent in Bosnia and Herzegovina and Serbia (28, 29, 31-33). Expenditure shares (5.14 percent for Nepal), on the other hand, exhibit limited variation, from 3.9 percent in India to 7.6 percent in Uganda (18, 29, 33, 34). Likewise, the regression estimates for unit value (0.78) and budget share (0.0066) also fall within the wide range reported in the literature, which varies across the member nations in the region and other LMICs (14, 17, 28, 29, 31, 33, 35, 36). So far as price elasticity of demand is concerned, global evidence suggests wide variation, exhibiting both elastic and inelastic demands. For example, Vietnam (-0.165) and Zambia (-0.2) exhibit inelastic demand, while Pakistan (-1.06), rural Bangladesh (-1.38), and Bosnia and

Herzegovina (-1.366) exhibit elastic demand. Most studies generally find inelastic demand for cigarettes (17, 33, 37, 39, 40, 41), but there is substantial cross-country variation in how smokers respond to changes in the price of cigarettes.

Policy relevance: Nepal's estimated value of price elasticity is relevant to the policymakers in the following ways. First, the estimates, together with their lower and upper limits, fall within the range of (-1, 0), suggesting that the cigarette consumption pattern in Nepal is relatively inelastic. This pattern aligns with the those reported in other countries in the South Asian region. This means that consumers reduce cigarette consumption by nearly 0.6 percent when the price increases by one percent. Therefore, a tax-increase policy on cigarettes, which raises the market prices, may reduce cigarette consumption to a lesser extent. This suggests that the government should combine the cigarette taxation policy with other measures (such as restrictions on smoking in public places, support for nicotine replacement therapies) to control cigarette consumption. Such a policy response can be expected in other settings where the demand for cigarette consumption is inelastic, such as in India and Bangladesh. Second, policymakers are also interested in the impact of taxation on cigarettes on revenue because it is directly affected by the consumer's demand response to price changes. Since the demand for cigarettes is relatively inelastic, an increase in excise tax could lead to higher revenue. With inelastic demand, consumers are less likely to reduce their consumption in response to a price increases, meaning that tax revenue could rise despite the higher prices of cigarettes. In Nepal, a recent policy discussion paper on excise tax on cigarettes in the 2024/25 budget examined the implications of tax increases on government revenue (22) The paper highlights that experience from the last three years demonstrates that increasing the tobacco taxes is an effective way to raise revenues.

Limitations: Our approach to estimating the price elasticity of demand for cigarettes is not without limitations. First, due to the low frequency of reported cigarette consumption, we used

repeated observations from a pool of three-year panel surveys to generate the price elasticity estimates. We corrected for the potential autocorrelation by year-fixed effects into the regression equations. This approach prevents the possible bias. Second, unlike many other studies, we were unable to report disaggregated estimates of the price elasticity by policy relevant variables due to the relatively low frequency of positive cigarette consumption within each cluster. Despite these limitations, our analysis provides a robust estimate of price elasticity that align with the existing literature in the South Asian context and current practices in using price elasticity estimates in the policy context of Nepal.

Future research directions: Based on our analysis, the demand of cigarette smoking will benefit from Depending upon the data availability, it may be possible to extend this study, where researchers may consider heterogeneity of the sample population for analysis (for example, based on income levels, rural-urban settings, age groups, and gender). This has the advantage of making tax and tobacco control policies more targeted and fairer. It is also plausible to collect data at frequent intervals and examine the smokers' behaviour, since this will help make informed and timely policy and behaviour-related decisions. Likewise, new surveys available in the region comprise a broader set of questions related to tobacco and cigarette smoking. It may be meaningful to utilize the datasets from these surveys to examine and make inter- and intra-regional comparisons of the shifts in the behavioural patterns of the smokers.

Acknowledgment: We sincerely thank the World Bank and all the individuals involved in collecting and making the panel data available, which has been invaluable for our research.

Competing Interest: None declared.

Disclaimers. The views expressed in the submitted article are his or her own and not an official position of the institution or funder.

Funding/Support: The authors received no financial support for this research.

References

1. WHO. Tobacco use falling: WHO urges countries to invest in helping more people to quit tobacco2021 2024-12-16. Available from: https://www.who.int/news/item/16-11-2021-tobacco-use-falling-who-urges-countries-to-invest-in-helping-more-people-to-quit-tobacco?utm_source=chatgpt.com.

2. Shariful Islam M, Rashid M, Sizear MI, Hassan R, Rahman M, Parvez SM, et al. Cigarette smoking and associated factors among men in five South Asian countries: A pooled analysis of nationally representative surveys. Plos one. 2022;17(11):e0277758.

3. West R. Tobacco smoking: Health impact, prevalence, correlates and interventions. Psychology & health. 2017;32(8):1018-36.

4. Burki TK. WHO releases latest report on the global tobacco epidemic. The Lancet Oncology. 2021;22(9):1217.

5. Anh PTH, Ross H, Anh NQ, Linh BN, Minh NT. Direct and indirect costs of smoking in Vietnam. Tobacco control. 2016;25(1):96-100.

6. John RM, Sung H-Y, Max WB, Ross H. Counting 15 million more poor in India, thanks to tobacco. Tobacco control. 2011;20(5):349-52.

7. Bobak M, Jha P, Nguyen S, Jarvis M. Poverty and smoking. Oxford University Press; 2000.

8. Gupta PC, Puntambekar N, Assunta M. South Asia’s evolving tobacco hydra: moving from quandary to hope. Tobacco Control. 2022;31(2):142-5.

9. WHO. Framework Convention on Tobacco Control. World Health Organization; 2003 2024-12-16.

10. Gravely S, Giovino GA, Craig L, Commar A, D'Espaignet ET, Schotte K, Fong GT. Implementation of key demand-reduction measures of the WHO Framework Convention on

Tobacco Control and change in smoking prevalence in 126 countries: an association study. The Lancet Public Health. 2017;2(4):e166-e74.

11. Rijo M. John HR, Blecher E. A rational taxation system of bidis and cigarettes to reduce smoking deaths in India. Economics for Health. 2011.

12. Jha P, Khan J, Mishra S, Gupta P. Raising taxes key to accelerate tobacco control in South Asia. bmj. 2017;357.

13. Deaton A. Quality, Quantity, and Spatial Variation of Price. The American Economic Review. 1988;78(3):418-30.

14. John RM. Price elasticity estimates for tobacco products in India. Health Policy Plan. 2008;23(3):200-9.

15. Joseph RA, Chaloupka FJ. The influence of prices on youth tobacco use in India. nicotine & tobacco research. 2014;16(Suppl_1):S24-S9.

16. Selvaraj S, Srivastava S, Karan A. Price elasticity of tobacco products among economic classes in India, 2011–2012. BMJ open. 2015;5(12):e008180.

17. Ahmed N, Mozumder TA, Hassan MT, Huque R. Demand for tobacco products in Bangladesh. Tobacco Control. 2022;31(6):737-43.

18. Huque R, Abdullah SM, Hossain MN, Nargis N. Price elasticity of cigarette smoking in Bangladesh: evidence from the Global Adult Tobacco Surveys (GATS). Tob Control. 2024;33(Suppl 2):s51-s8.

19. WHO. Tobacco Retail Price Data 2024 [updated 2024-12-16. Available from: https://apps.who.int/gho/data/view.main.TOBRETAILv.

20. Economics for Health. South Asia Tobacco Control Report. 2022 2024-12-16.

21. Organization WH. Tobacco control: Raise taxes on tobacco2025. Available from: https://www.who.int/data/gho/data/themes/topics/raise-taxes-on-tobacco.

22. National Development Research Institute. Taxing Tobacco in Nepal: What can be done. National Development Research Institute; 2020.

23. National Development Research Institute. Policy Paper: Excise Tax on Cigarettes in the 2024/25 Budget. Kathmandu, Nepal: National Development Research Institute,; 2024.

24. Wickham H, Averick M, Bryan J, Chang W, McGowan LDA, François R, et al. Welcome to the Tidyverse. Journal of open source software. 2019;4(43):1686.

25. Berge L, Krantz S, McDermott G, Lenth R. Package 'fixest'. 2021.

26. Lumley T, Lumley MT. The survey package. hospital. 2007;24:1.

27. Canty A, Ripley B. Package 'boot'. Bootstrap Functions CRAN R Proj. 2017.

28. Walker T, Kawasoe Y, Shrestha J. Risk and vulnerability in Nepal: Findings from the household risk and vulnerability survey: World Bank; 2019.

29. Vladisavljevic M, Zubović J, Đukić M, Jovanović O. Tobacco price elasticity in Serbia: evidence from a middle-income country with high prevalence and low tobacco prices. Tobacco control. 2020;29(Suppl 5):s331-s6.

30. Chelwa G, van Walbeek C. Does cigarette demand respond to price increases in Uganda? Price elasticity estimates using the Uganda National Panel Survey and Deaton's method. BMJ open. 2019;9(3):e026150.

31. NRB. Current Macroeconomic and Financial Situation Tables. Nepal Rastra Bank; 2018 2024-12-16.

32. Adeniji F. Consumption function and price elasticity of tobacco demand in Nigeria. Tobacco Prevention & Cessation. 2019;5.

33. Perera KMN, Guruge GD, Jayawardana PL. Household expenditure on tobacco consumption in a poverty-stricken rural district in Sri Lanka. Asia Pacific Journal of Public Health. 2017;29(2):140-8.

34. Gligorić D, Pepić A, Petković S, Ateljević J, Vukojević B. Price elasticity of demand for cigarettes in Bosnia and Herzegovina: microdata analysis. Tobacco Control. 2020;29(Suppl 5):s304-s9.

35. NSO India. Household Consumption Expenditure Survey, 2022-2023. National Statistical Office, Government of India; 2023.

6. Supplementary Material or Appendices

Not Available

Title

Price elasticity of demand for cigarettes in Nepal: evidence from a lower middle-income country in South Asia using Deaton's demand model

Authors Information

1. **Name:** Vishnu Prasad Sapkota;

   Affiliation: Department of Economics, Tribhuvan University, Nepal Commerce Campus, Min Bhavan Marga, Kathmandu, Nepal.

   Email address: vishnu.sapkota@ncc.tu.edu.np; visapkota@gmail.com

   ORCID ID: https://orcid.org/0000-0002-4014-8267

2. **Name:** Umesh Prasad Bhusal;

   Affiliation: Health Economics Researcher, Kathmandu Nepal;Nepal.

   Email Address: umbhusal2020@gmail.com;

   ORCID ID: https://orcid.org/0000-0001-9331-6028

3. **Name:** Nirmal Kumar Raut;

   Affiliation: Central Department of Economics; Central Campus, Tribhuvan University, Kirtipur, Kathmandu, Nepal;Nepal.

   Email address: nirmal.raut@cdec.tu.edu.np;

   ORCID ID: https://orcid.org/0000-0002-4234-4739

Corresponding author

1. **Name:** Vishnu Prasad Sapkota;

   Affiliation: Department of Economics, Tribhuvan University, Nepal Commerce Campus, Min Bhavan Marga, Kathmandu, Nepal.

   Email address: vishnu.sapkota@ncc.tu.edu.np; visapkota@gmail.com

   ORCID ID: https://orcid.org/0000-0002-4014-8267

Abstract

Background: The prevalence of cigarette smoking is declining globally; however, it remains highest in South Asia. Governments in this region rely on taxation as an instrument to reduce cigarette demand. The effectiveness of such instruments depends on consumers' underlying preferences and behavioural responses to changes in price – it is commonly expressed in terms of price elasticity of demand. A few studies in this region used ~~widely~~ available cross-sectional and panel surveys to estimate cigarette price elasticity using Deaton's demand model. In this context, we aim to estimate the price elasticity of cigarettes in Nepal.

Methods: We applied Deaton's demand model to estimate the cigarette price elasticity using a three-year panel microdata (2016-2018) from the Nepal Household Risk and Vulnerability Survey. We used ~~5653 households observed over three waves; the study units reported~~ data from 5,653 households observed over three waves; the study units reported the purchased quantities of cigarettes and related expenditures. Deaton's demand model exploits spatial variation in unit values of cigarettes to measure price elasticity. We adjusted the basic model for possible bias that may arise from panel nature of the data.

Results: We found that the estimate of price elasticity for cigarette demand was -0.58 with [95% CI: -0.79 ~~– 0.37], and~~ -0.37], and the expenditure elasticity estimate was 1.06 [95% CI: 0.54 – 1.57]. These statistics indicate that the cigarette demand is relatively inelastic to price, and unitary elastic to income.

Conclusions: The negative price elasticity estimate for Nepal indicates that the increase in excise taxes can be effective both in reducing cigarette consumption and raising revenues. This also provides additional evidence that the routine surveys, including panel data, that are

designed to monitor living standards can be valuable to estimate price elasticity using the Deaton's method.

Keywords

Price elasticity; Cigarette consumption; Taxation; Tobacco industry; Tobacco policy.

Highlights

What is already known about the topic?

Very few countries in ~~the South Asia have~~ South Asia have a price elasticity estimate for cigarette consumption. Those ~~that~~ who report the figure, the responsiveness of cigarette consumption to prices is inelastic in this region, specifically in India and Bangladesh. Such an estimate is not available for Nepal.

What this study adds?

This is the first study ~~that has estimated the responsiveness of price changes to cigarette consumption in Nepal using a~~ to estimate the responsiveness of price changes to cigarette consumption in Nepal using panel survey data. The estimated price elasticity was -0.58 with [95% CI: -0.79 ~~– 0.37], and~~ -0.37], and the expenditure elasticity estimate was 1.06 [95% CI: 0.54 – 1.57].

How this study might affect research, practice or policy?

The negative price elasticity estimate for Nepal ~~indicates that the increase in excise taxes can be effective both in reducing cigarette consumption and raising~~suggests that increasing excise taxes can be effective in reducing cigarette consumption and increasing tax revenues for governments. This also provides additional evidence that the panel ~~routine~~ surveys that are

designed to monitor living standards can be valuable to estimate price elasticity using the Deaton's method.

1. INTRODUCTION

Cigarette smoking is declining globally; however, its prevalence remains highest in South Asia. Recent evidence ~~report~~ reports that 27.1% of people aged 15 and above in South Asia ~~smokes~~ smoke cigarettes, with Nepal reporting an even higher rate of 30.4%, compared to the global average of 22.3% (1, 2). Cigarette smoking is ~~documented as one of the important~~ a major modifiable risk-factors for leading non-communicable diseases (3, 4). Beyond its impact on health, smoking ~~further aggravates~~worsens living standards and poverty (5-7). These ~~widely reported~~ health and economic impacts, together with the transnational nature of the tobacco industry in the South Asian region, pose multifaced challenges for tobacco control (8). The Framework Convention on Tobacco Control (FCTC) recommends demand reduction of tobacco consumption as an important theme and suggests ~~an array of~~ interventions such as taxation, health warnings, and advertising bans, to ~~make progress in~~advance this ~~dimension~~ agenda (9).

Taxation is a widely employed policy instrument to reduce ~~the demand for the~~ tobacco consumption, including cigarette smoking (10). ~~Among the countries I~~In South Asia, ~~taxation policies~~it ~~have the potential to realize~~ can achieve dual goals of revenue mobilization and ~~improving~~ improved public health ~~(~~by reducing cigarette demand ~~)~~(11, 12)~~;~~. H~~h~~owever, ~~the~~ its effectiveness ~~of such policy interventions~~ depends on consumer responses to price changes ~~in price~~. Price elasticity of demand, ~~is widely used~~a common measure ~~to evaluate such behavioural~~of these response~~s.~~s, ~~It measures~~shows how consumers adjust their purchasing behaviour ~~in response~~ to price changes, ~~and this adjustment is fundamentally rooted in~~ reflecting ~~their~~ preferences, ~~including the~~ perceived value, utility, or necessity of the product.

In the literature, the price elasticity of demand for cigarette consumption is measured based on time series and cross-sectional data. The latter is commonly used by most low- and middle-income countries employing Deaton's method (13) ~~method~~. This method provides a ~~theory and~~

related procedureframework to estimate the price elasticity of demand for regular commodities when long-term time series data are challenging to obtainunavaiialable or incomplete. The methodIt provides a robust framework to quantify price responsiveness while uncovering the behavioural factors underlying purchasing decisions. The methodIt uses rich data from large-scale cross-sectional surveys, primarily designed to monitor living standards and consumer prices. –By framing price elasticity as an outcome of consumer preferences and price responsiveness, this study not only quantifies the sensitivity of cigarette demand to price changes but also situates these findings within the broader context of consumer decision-making.

The price elasticity of cigarette demand in Nepal holds significant importance and presents a compelling case study in South Asia for the at least fivefollowing reasons. First, cigarette smoking preferences are similar among the adults population in South Asian countries (8), and this is directly reflected in price elasticity estimates. These countries haveyet rich studies using Deaton's method remain scant despite the availability of rich microdata on tobacco consumption, including cigarette smoking; however, studies that estimate the price elasticity of demand for cigarettes in South Asia using Deaton's method are scant. In Nepal, Tthis is the first study in Nepal to estimate the price elasticity of cigarette demand employing the Deaton's method (13) method. Regional evidence is limited and mixed: iIn India, earlier estimates showed a lower price elasticity of demand (less than -0.20) for cigarettes (-0.179) compared to *bidis* and bidis and leaf tobaccoother smokeless tobacco (-0.87) (SLTs), such as leaf tobacco(14),. while sSubsequent studies showed an increase in the price elasticity of demand for cigarettes, ranging from -0.2645 (for the wealthiest group) to -0.832 (for the poorest group) (15, 16), though most are outdated or limited to subgroups such as youth. These studies either provide elasticity estimates that are representative of a small subgroup of the population, such as the youth age group, or are outdated by more than a decade. -Studies in Bangladesh and

Pakistan ~~measure the price elasticity using~~rely on recent surveys (17, 18), but~~.~~ evidence from other South Asian countries remains scarce~~Besides these studies, there appears to be limited evidence of the price elasticity of cigarette consumption from other South Asian countries~~. Second, cigarette smoking prevalence in South Asia is higher ~~in this region~~ compared to the global average, with diverse products and ~~also occupies a unique place because of the broad spectrum of cigarettes consumed and the nature~~ strong ~~of~~ transnational industry presence ~~companies engaged in cigarette production in the region~~ (8). Third, ~~the~~ cigarette prices in purchasing power parity (PPP) $ ~~for the mostly bought cigarette products in Nepal is 7.85, comparable to South Asian countries~~ are broadly comparable across the region (7.85 in Nepal, 8.66 in India, 11.96 in ~~Maldeevs~~Maldives, and 2.91 in ~~Bandladesh~~Bangladesh) (19),~~.~~ and ~~Similarly, the total~~ tax rates ~~in Nepal~~ are similar~~that covers specific excise, ad valorem excise, and value-added tax per cigarette is comparable to South Asian countries(19). In addition~~, yet no ~~South Asian~~ country~~ies~~ met the WHO-recommended ~~level of~~ 75% ~~tax~~ share of the retail price in 2022 (20).

Table 1 shows the trend of cigarette prices and taxes in Nepal over the period of 10 years. The cigarette prices have been increasing at constant rate of ~1.00 USD (in PPP) each year between 2012 to 2022. This means that the household face a reasonably constant increment in prices before and during the survey period. During the same period, the excise taxes have remained stable between 26 – 30 percent of cigarette prices. These statistics point to the fact that price changes and tax on cigarette were relatively stable during the study period.

Table 1: Cigarette price and tax in Nepal between 2012 and 2022.

**Year**	**2012**	**2014**	**2016**	**2018**	**2020**	**2022**
Retail price ~~of cigarette~~ (per 20 sticks in PPP dollars)	3.65	4.69	5.63	6.78	8.38	10.43

Formatted: Space After: 0 pt

Formatted: Right, Line spacing: Double

Formatted: Font: Bold

Formatted: Line spacing: Double

Formatted Table

Formatted: Line spacing: Double

Formatted: Strikethrough

Taxes as percentage of cigarette price (%)	29.86	27.79	26	30	27	31.39
Source (21): https://www.who.int/data/gho/data/themes/topics/raise-taxes-on-tobacco						

Commented [UB1]: Can we just say "Tax rate (ad valorem)"?. Please note GoN impose tax per 1000 sticks

Formatted: Line spacing: Double

Formatted: Line spacing: Double

~~In Nepal, cigarette excise taxes have increased across tiers in recent years. For example, on the most consumed tier (75mm to 85mm in length filtered) the rate rose from NPR 1,920 per 1,000 sticks in 2020 to NPR 2,970 in 2023 (NPR 2,400 in 2021 and NPR 2,880 in 2022), leading to modest increases in retail prices. The total tax share of the retail price is currently around 41%.~~ Fourth, the cigarette industry in Nepal ~~is an important source of~~contributes significantly to public revenue ~~, occupying significant share of the tobacco industry, and enjoys~~and receives ~~stronger~~ government support, partly due to concerns over~~for example, a slow increase in tax on tobacco products — it is considered reasonable in light of the potential decrease in~~ employment and investment ~~in this sector~~ (8, 22). Fifth, although ~~the tax base of tobacco in Nepal, including South Asia, is heavily dependent on tobacco users who smoke cigarettes. N~~non-cigarette tobacco products account for more than 60% of the tobacco consumption, ~~in all countries in this region~~, ~~yet~~ they contribute less than 20% of the total tobacco tax revenue (8, 23).

In this context, we aim to estimate the ~~cigarette~~ price elasticity of cigarette demand in Nepal using ~~Deaton (13)~~ the demand model of Deaton (13). The approach outlines the ~~related~~ theory and procedure to estimate the price elasticity of demand for regular commodities from cross-sectional surveys,~~;~~ ~~these surveys~~which are often used to monitor poverty and living standards in low-and middle-income countries. In these surveys, households report both expenditure and physical quantities of cigarettes, ~~both of~~ which are affected by the market prices ~~of cigarettes~~. The two reported variables can be used to derive the unit values by dividing ~~one~~ expenditure by ~~the other~~quantity. However, these unit values cannot be treated as proxies ~~of~~ for prices because they are affected by measurement error and quality effects. These surveys ~~designs~~

observe clusters of households simultaneously, where there is no genuine variation in market prices, but ~~there can be~~ variation can exist ~~in prices~~ between ~~the~~ clusters. The Deaton method exploits this variation to correct the measurement error and quality effects to arrive at the price elasticity of cigarettes. In the next section, we elaborate on the correction method in detail.

2. METHODOLOGY

In this section, we first elaborate on Deaton's approach to measure the price elasticity of demand for regular commodities. Then, we apply the method to estimate the demand equation for cigarette consumption using panel survey ~~microdata~~ from Nepal. In addition to this, we also elaborate additional assumptions and the adjustments we made in the original model that was applied to cross-sectional surveys. Besides this, we explain the data source, clusters, and variable definitions.

Specification and estimation

~~W~~We used the information on reported quantities (q_{ht}) ~~–~~and expenditure (c_{ht}) on cigarettes available in the household survey ~~microdata~~ over the three waves. These values reflect that households ~~in a given~~in each locality face common market prices in each wave. However, the information on cigarette prices is not ~~un~~available in the dataset. In this context, the proposed method exploits the commonality of prices ~~in a given~~in each locality, which is reflected implicitly in q_{ht} and unit values of the cigarettes ~~:~~ $(v_{ht} = c_{ht}/q_{ht})$. In Deaton's model, the key identifying assumption is that inter-cluster variation in prices serves as an instrumental variable (IV) for demand. This assumption remains intact in our panel context, where households face the same cluster price at each time point, but with possible temporal variation. Such variation requires careful treatment in unit values and budget share regressions (equation 1 and 2) but does not invalidate the basic identification and estimation strategy, which we discuss below.

Formatted: Space After: 8 pt, Don't keep with next, Border: Top: (No border), Bottom: (No border), Left: (No border), Right: (No border), Between : (No border)

With this information and underlying assumptions, we Deaton (13) suggested to estimated unit values (v_{ht}) and budget-share (w_{ht}) regression using the following specifications,

$$ln\ v_{hct}\ =\ \alpha^1 + \beta^1 ln\ x_{hct}\ + Z_{hct}\gamma^1 + \psi ln\pi_c + y_t + u_{hct}^1 \qquad 1$$

$$w_{hct} =\ \alpha^o + \beta^o ln\ x_{hct}\ + Z_{hct}\gamma^o + \theta ln\pi_c + f_c + y_t + u_{hct}^o \qquad 2$$

Where, $ln\ v_{hct}$ is the log of unit values derived in the previous step, w_{hct} represents the budget-share for cigarette consumption: $w_{hct} = c_{hct}/x_{hct}$. $ln\ x_{hct}$ is a log of total consumption for the household, and Z_{hct} includes a series of household-specific characteristics such as household size, age and gender composition, household head's characteristics. These variables shift unit values and budget share. In addition to this, the specification also includes consumption quintiles and year dummies in Z_{hc}. These variables are measured for each household h living in cluster c. The f_c is the cluster-specific fixed-effects, y_t is year specific fixed effect, and u_{hct}^1 and u_{hct}^o are error terms in the respective equations. Deaton in his original set-up assumed that Tthese error terms also incorporate the measurement errors in the unit values and budget share. Another important aspect is that equation 1 does not include cluster fixed effects because Deaton (13) elaborates that conditional on cross-sectional and temporal variation in prices, unit values depend only on quality effects and measurement errors. $ln\ \pi_c$ appears in two equations, which are unobserved prices and are constant within the clusters because all the households within the cluster face a common price for the cigarette after removing year specific fixed effects. We estimated the two equations without $ln\ \pi_c$ and, later, recovered the associated coefficients from the expressions proposed by Deaton (13) in their

theoretical paper. The relationship between u^1_{hct} and u^o_{hct} was used to correct the price elasticity of demand for cigarettes.

In the pooled OLS set-up of equations 1 and 2, we adjust for possible bias that may arise from repeated measurements over three waves, possibly due to temporal trends in unit values and budget shares, temporal correlation in the error terms of the respective equations, and unobserved changes in cigarette consumption patterns that may be correlated with the covariates. We correct for these as follows:

1. We add year-specific fixed effects to ensure that between-cluster variation in the unit value and budget share regressions is free from temporal trends. This ensures that the basic requirement to apply the Deaton demand model is maintained.

2. In equation 1 and 2, within-cluster variation may be due to (i) variation in cigarette consumption preferences over the three-year period, (ii) autocorrelation in the error terms in equations 1 and 2, and (iii) measurement error (correlation between the error terms of equations 1 and 2). Of these, (iii) is key to Deaton's correction strategy for measurement error. We minimize bias that may result from (i) and (ii) with the following adjustments and assumptions:

   - (24)We adjust for temporal correlation in u^1_{hct} and u^o_{hct} by allowing the errors to cluster over time while estimating the measurement error.

   - We assume that policy-driven shifts in cigarette preferences are minimal during the survey period – a stable, marginal change in cigarette prices and taxes between 2016–2018 (shown in Table 1) provides evidence that this assumption is met.

   - Cigarette consumption behaviour exhibits a stable pattern over short time interval. A one-year gap between each survey wave makes preference changes unlikely –

Formatted: Font: (Default) Times New Roman, 12 pt
Formatted: Font: 12 pt
Formatted: Font: 12 pt
Formatted: Font: 12 pt
Formatted: Font: (Default) Times New Roman, 12 pt
Formatted: Font: 12 pt
Formatted: Font: 12 pt
Formatted: Font: 12 pt
Formatted: Font: (Default) Times New Roman, 12 pt

longitudinal studies show that transitions between smoker and non-smoker states are rare within one year (24).

Together, with these assumptions and adjustments, the coefficient estimates from the pooled OLS and measurement error corrections still maintain the properties of the original Deaton (1988) model.

Next, we derived demand for cigarettes and unit values at the cluster level after removing the effects of household consumption, ~~and other~~ socio-demographic and household head's characteristics and year specific fixed effects using equations 1 and 2. These values were then averaged over the clusters because our primary interest was to estimate the elasticity at the cluster-level unit values ($\hat{y}_c^1$) and budget share ($\hat{y}_c^o$) values. These values carry the commonality of price and purchases of cigarettes in the given cluster. From equations (3) and (4) onwards, the analysis relies on cluster-level averages of unit values and budget shares, after purging household characteristics and year effects. At this stage, our procedure is identical to Deaton's cross-sectional approach.

$$\hat{y}_c^1 = \frac{1}{n_c^+} \Sigma_{h=1}^{n_c^+} \left(ln v_{hc} - \hat{\beta}^1 \, ln \, ln \, x_{hc} \; - \; Z_{hc} \hat{\gamma}^1 \right) \quad 3$$

$$\hat{y}_c^0 = \frac{1}{n_c} \Sigma_{h=1}^{n_c} \left(w_{hc} - \hat{\beta}^o \, ln \, ln \, x_{hc} \; - \; Z_{hc} \hat{\gamma}^o \right) \quad 4$$

Based on the cluster-level values derived in the previous step, we estimated a regression coefficient of cluster-level demand ($\hat{y}_c^o$) on cluster level unit values ($\hat{y}_c^1$) based on the expression in equation 5 – it was a standard errors-in-variables regression, where we corrected measurement errors using the covariance and variance of errors. In this step, we allowed the households to be correlated across the time to correct the autocorrelation in error terms in equation 1 and 2.

$$\hat{\phi} = \frac{\left(cov(\hat{y}_c^1, \hat{y}_c^o) - \frac{\hat{\sigma}^{10}}{n_c}\right)}{\left(var(\hat{y}_c^1) - \frac{\hat{\sigma}^{11}}{n_c^+}\right)} \qquad 5$$

In the final step, we used a quality correction formula derived by Deaton (13) to obtain the price elasticity estimates of cigarette demand ($\hat{\varepsilon}_p$).

$$\hat{\varepsilon}_p = \left(\frac{\hat{\theta}}{\bar{w}}\right) - \hat{\psi} \qquad 6$$

In equation 6, $\bar{w}$is the sample mean of the share of total household consumption expenditure on cigarettes. $\hat{\psi}$ and $\hat{\theta}$ are of the coefficients on the unobserved cluster-level cigarette prices in equations ~~3.2~~1 and ~~3.3~~2, respectively. We recovered these values using the following expressions proposed by Deaton (1988).

$$\hat{\psi} = 1 - \frac{\hat{\beta}^1(\bar{w}\underline{w} - \hat{\theta})}{\hat{\beta}^1 + \bar{w}} \quad 7; \qquad \hat{\theta} = \frac{\hat{\phi}}{1 + (\bar{w} - \hat{\phi})\hat{\zeta}} \quad 8; \qquad \hat{\zeta} = \frac{\hat{\beta}^1}{\hat{\beta}^o + \bar{w}(1 - \hat{\beta}^1)} \quad 9$$

We also recovered the expenditure elasticity of demand using the following expression as suggested by Deaton (1988),

$$\widehat{\varepsilon_I} = 1 + \left(\frac{\hat{\beta}^o}{\bar{w}}\right) - \hat{\beta}^1 \qquad 10$$

We used R-programming for statistical computing for data management and analysis. In this process, we use the following R packages: *tidyverse* for data management, *fixest* for fixed effect regression models, *survey* to produce survey-weighted estimates, and *boot* to estimate bootstrap standard errors (25-28).

Survey data, cluster definitions, and measurement

We used data from the Nepal Household Risk and Vulnerability Survey – a three-year (2016-2018) household panel. The data was accessed in July, 2022 from The World Bank data

repository[1]. The details about the survey are available in Walker et al. (2019) (29). The survey instrument was based on the World Bank's Living Standards Measurement Study which covered information on socio-demographics, consumption expenditure, income, and labor supply. The sampling frame was all households in rural municipalities, municipalities and sub-metropolitan areas of Nepal. Households were stratified by 11 regions. In the first stage of sampling, 400 wards were randomly selected, with the number within each region predetermined by that region's population share. In the next stage, 15 households were randomly selected within each sampled ward, giving a total of 6,000 sampled households interviewed in 2016 and targeted for reinterview in 2017 and 2018. Attrition was low: 5835 (97.3%) households from the initial sample were reinterviewed in 2017, and 5,696 (94.9%) were reinterviewed in 2018. A total of 5,654 (94.2%) households were interviewed in all three waves. We dropped one household that did not report food expenditure in 2017 and used a balanced, complete-cases sample of 5,653 households, forming 16,959 observations for the analysis.

In this analysis, we use clusters defined by the survey design. This allowed a total of 400 clusters in our analysis. Altogether, in the three waves, there were 42.4 observations in each cluster, which resulted from a follow-up of each household three times in 2016-2018. This approach is in sharp contrast to the approach followed by others Vladisavljevic, Zubović, Đukić and Jovanović in Serbia (30) where clusters are defined as interaction of $clusters \times waves$. Similarly, Chelwa and van Walbeek in Uganda (31) conducted separately analyses for each wave. However, we used repeated observations in the clusters for the following reasons: 1) this increased the cluster sizes, increasing the robustness of the estimates; 2) this increased the reliability of computations of unit values because each household reports the consumption three

[1] https://microdata.worldbank.org/index.php/catalog/3705

times; and 3) this increased the efficiency of the estimates by reducing the error variance. There is the possibility of autocorrelation and year-specific effects in the errors in equations 1 and 2, which we addressed by adjusting the regression equations with year-specific fixed effects and clustering of error terms u_{hc}^{1} and u_{hc}^{o} in equations 1, 2, and 5. This minimized the possibility of bias resulting from the autocorrelation.

The survey questionnaire provides information about the important variables used in the analysis. We used weekly household expenditure on cigarettes, number of cigarettes purchased, and total annual household expenditure – the latter was converted into weekly expenditure to express all the estimates in a common recall period. The monetary values were adjusted to 2017 prices by using the Consumer Price Index available from Nepal Rastra Bank (32). Besides this, as discussed in the equation 1 and 2, we used other socio-demographic variables at the household level – these include household size, age and gender composition of the household members, number of male members, education and gender of household head, maximum education of household members, consumption quintile and place of residence. These variables were recovered from section 1 and section 2 of the survey questionnaire, except for the consumption quintiles, which were based on total annual per capita consumption expenditure. The place of residence of the household was not initially available in the questionnaire – we defined rural households as those living in rural municipalities and urban households as those living in municipalities and sub-metropolitan cities.

3. RESULTS

Table ~~1~~2 provides the descriptive statistics of the variables used to estimate equations 1 and 2. We found that nearly 17% of households, pooled over three years, reported cigarette consumption in this survey. Regarding expenditure estimates, total household consumption expenditure was 3,562.7 NPR per week, and 15% of households reported non-zero expenditure

on cigarettes. On average, households reported a cigarette consumption of 42.3 pieces per week and spent on average about 170 NPR. Based on this, the unit value of cigarettes per piece was 8.2 NPR, reflecting changes in cigarette prices and choices of cigarette quality purchased on the local market. The share of cigarette spending relative to total household consumption was 5%.

Table 21: Summary statistics of key outcome variables

**Variables**	**Observations**	**Mean**	**SD**	**Q10**	**Q90**
Total consumption per week (NPR)	16959	3562.7	2514.4	1554.9	6056.4
Positive expenses on cigarettes	16959	0.1530	0.3600	0.00	1.00
Cigarette quantities per week (pieces)	2756	42.3	42.0	7.0	120.0
Expenditure on cigarettes per week (NPR)	2756	169.9	342.2	40.00	350.0
Unit value of cigarette (NPR)	2756	8.2	15.9	1.8	10.0
Budget share of cigarette	2756	0.0514	0.0951	0.0118	0.1030
Number of observations in each cluster		42.4	3.1	39	45
Number of households in each cluster		14.1	1.0	13	15
Number of clusters	400				
Number of clusters X years	1200				

Notes: The statistics are pooled over three survey years. Survey weights are used. SD = standard deviation. Q10 and Q90 are quantiles at 10% and 90% points, respectively. NPR = Nepalese Rupee, 2017 prices, NPR1000 = US$9.64.

Table 32 presents the descriptive statistics of the variables used as controls in the regression equation 1 and 2. Again, the statistics were pooled over three surveys. The average household size is about 5, and average number of male members is 2.4. We reported the maximum education of household members irrespective of the member's age – the statistics stand at 7 years. We also reported average percentage of members by age and sex – 0 to 5 years (4% male and 3.5% female), 18 to 49 years (13% male and 25% female), and 50 years or older (11% for

both male and female). Regarding household head characteristics, 21% of household heads were female, and had four years of education on average.

Table 3~~2~~: Summary statistics of the control variables

Variables	Observations	Mean	SD	Minimum	Maximum
Household demographics					
Household size	16959	4.9711	2.0328	1	22.0000
Number of male members	16959	2.3617	1.2760	0	10.0000
Maximum education of HH members (years)	16959	6.9137	4.5758	0	15.0000
Proportion of household members ~~(age in years)~~					
Male [0-5] years	16959	0.0406	0.0894	0	0.6667
Female [0-5] years	16959	0.0364	0.0869	0	0.6667
Male [6-17] years	16959	0.1313	0.1627	0	0.8000
Female [6-17] years	16959	0.1212	0.1585	0	1.0000
Male [18-49] years	16959	0.1840	0.1627	0	1.0000
Female [18-49] years	16959	0.2512	0.1550	0	1.0000
Male [>49] years	16959	0.1185	0.1549	0	1.0000
Female [>49] years	16959	0.1169	0.1659	0	1.0000
Household head's characteristics					
HH head (female) (=1)	16959	0.2115	0.4084	0	1.0000
HH head's education (years)	16959	4.0358	4.3579	0	15.0000

Note~~s~~: The statistics are pooled over three years. Survey weights are used. SD = standard deviation. HH= household

Table 4~~3~~ ~~shows estimated results from the first stage regression for unit value and budget share as per equations~~presents the estimated results from the first-stage regression for unit value and budget share, as per Equations 1 and 2. In column (1), we show the estimated coefficients from unit value regression, which is a conditional model because it was estimated on the households that report cigarette consumption. In this model, the coefficient for total expenditure is positive and statistically significant; it is a measure of the quality elasticity of expenditure, which is about 0.78. Similarly, the coefficient for household size is negative, indicating a lower unit

value with the increase in household size. However, we do not find statistically significant effects of household socio-demographic composition and the household head's characteristics on unit value. If we ignore the statistical significance, the coefficients are of ~~expected sign; for example, an increase in the percentage of male members has negative effects~~the expected sign; for example, an increase in the percentage of male members has a negative effect on the unit values of cigarettes. Similarly, statistically significant and negative coefficients for quintiles indicate that relative to the lowest quintile, households belonging to all other quintiles use cigarettes with lower unit values. In addition to this, concerning ~~survey year, we do not find any statistically significant changes in unit values even though~~ the survey year, we do not find any statistically significant changes in unit values, even though the coefficients are relatively large and positive. Finally, cluster fixed effects are jointly statistically significant, confirming pronounced spatial variation in unit values.

In column (2), we present coefficients associated with the budget share regression, which is an unconditional model, as the model was estimated on the whole sample irrespective of whether households reported cigarette consumption. The coefficient on the log of total consumption is positive and statistically significant, indicating that households with higher consumption expenditure tend to allocate more budget for cigarettes – a one percent increase in total consumption increases the budget share by 0.6%. Similarly, the coefficient on household size is negative, although not statistically significant. Besides this, we find statistically significant coefficients for the quintiles – relative to the baseline (lowest quintile), the higher levels of quintiles have negative and statistically significant coefficients~~ indicating that relatively richer households tend to spend less percentage of~~ , indicating that relatively richer households tend to spend a smaller percentage of their household total consumption on cigarettes. Similarly, the budget share tends to decrease over time – relative to 2016, the coefficients for 2017 and 2018

are negative and statistically significant. Finally, cluster fixed effects are jointly statistically significant, confirming the spatial variation in budget share.

Table 43: Unit-value and budget share regressions for cigarette consumption in Nepal

Variables	(1) Unit values of cigarettes in logs	(2) Budget share for cigarettes
Total consumption in logs	0.7785** (0.1709)	0.0066* (0.0021)
Household size in logs	-0.5774** (0.1318)	-0.0098 (0.0053)
(Household size in logs)^2	-0.0059 (0.0739)	0.0018 (0.0022)
**Household members' composition (in percent)**		
Males [0-5] years	-0.2599 (0.2319)	-0.0133* (0.0045)
Males [6-17] years	-0.1524 (0.1584)	-0.0047 (0.0070)
Males [18-49] years	-0.1428 (0.0978)	-0.0015 (0.0067)
Males [>49] years	-0.0529 (0.3726)	0.0020 (0.0055)
Females [0-5] years	0.1740 (0.2946)	-0.0043 (0.0089)
Females [6-17] years	-0.0632 (0.1095)	-0.0066 (0.0036)
Females [18-49] years	0.0757 (0.1063)	-0.0051** (0.0010)
**Household head's characteristics**		
HH head (female) (=1)	0.0624 (0.1218)	-0.0035 (0.0017)
HH head's education (years) in logs	0.0229 (0.1260)	0.0005 (0.0003)
(HH head's education (years) in logs)^2	0.0169 (0.0601)	-0.0004* (0.0001)
**Other household characteristics**		
Number of male members	0.0317 (0.0146)	-0.0007 (0.0013)
Maximum education of HH members (years) in logs	-0.0307 (0.1430)	0.0016 (0.0013)
(Maximum education of HH members (years) in logs)^2	-0.0018 (0.0553)	-0.0012 (0.0005)
**Consumption quintiles (First – baseline)**		
Second (=1)	-0.2475** (0.0424)	-0.0025* (0.0008)
Third (=1)	-0.4552* (0.1165)	-0.0048* (0.0015)
Fourth (=1)	-0.5647* (0.1313)	-0.0060 (0.0020)
Fifth (=1)	-0.9139** (0.2115)	-0.0089* (0.0026)
**Place of residence (Rural – baseline)**		
Urban (=1)	-0.0931 (0.1093)	-0.0018 (0.0006)
**Survey year (2016 - baseline)**		
Year - 2017 (=1)	0.1021 (0.0367)	-0.0030*** (0.0001)
Year - 2018 (=1)	0.0800 (0.0871)	-0.0039*** (0.0003)

Variables	(1) Unit values of cigarettes in logs	(2) Budget share for cigarettes
**Cluster dummies**	**F (377, 2378)**	**F (911, 1844)**
	**2.9958*** **	**1.7631*** **
**R^2 Adjusted**	**0.262**	**0.037**
**Observations**	**2756**	**16959**

Notes:. OLS estimates of coefficients from models 1 and 2. Standard errors (SEs) in parentheses. The autocorrelation of errors over the years is adjusted. The summary of controls is covered in Table 2. The analysis is based on a panel of 5,653 households observed over three years (2016-2018), giving 16,959 observations. Sample and specification in equations 1 and 2. NPR = Nepalese Rupee, 2017 prices, NPR1000 = US$9.64. HH=household. *, **, *** indicate statistical significance at 10%, 5%, and 1%, respectively.

Table 45 provides summary of the expenditure and price elasticity estimates derived from the procedure described in equations 3 to 10. We use the coefficients together with the key parameters from equation equations 1 and 2 to find the expenditure elasticity ($\widehat{\varepsilon_I}$)—the estimated value was 1.06, a positive, statistically significant value that means that among the households consuming cigarettes, a 10% higher total household expenditure is associated with an increase in the quantity of cigarettes smoked by nearly the same amount.

Table 54: Elasticity estimates of cigarette demand

	Term	Estimate	SEs	P-values	Confidence interval (95%) Lower	Confidence interval (95%) Higher
(1)	Expenditure elasticity ($\widehat{\varepsilon_I}$)	1.0578	(0.2619)	<0.001	0.5445	1.5712
(2)	Price elasticity* ($\widehat{\varepsilon_p}$)	-0.5759	(0.1073)	<0.001	-0.7949	-0.3745

Notes: Row (1) provides expenditure elasticity using equation 10. Row (2) provides the price elasticity of cigarette demand using equations 6-9. Standard errors (SEs) in parentheses. SEs for price elasticity are based on 1,000 bootstrap replications from equations 6-9 and SEs for expenditure elasticity are based on 1,000 bootstrap replications of the equations 1, 2 and 10.

*We also undertake robustness checks of $\widehat{\varepsilon_p}$ across the three survey panels – the estimates range from -0.15 in 2016 to -0.95 in 2018.

After estimating the expenditure elasticity and, together with the estimates from cluster-level averages of budget share and unit values, we estimate the price elasticities as elaborated in equations 3 – 6, and the results are shown below in row (2) of Table ~~4~~5. The estimated value of price elasticity ($\hat{\varepsilon_p}$) of cigarettes was -0.58, which indicates that if cigarette prices are increased by 10%, the corresponding demand will fall only by 5.8%. We computed a 95% confidence interval based on 1,000 bootstrap replications, and the lower and upper intervals are -0.79 and -0.37, respectively.

4. DISCUSSION AND CONCLUSION

In this paper, o~~We presented the analysis in this paper with the two aims. First, we~~ur objective is to estimate~~ed~~ the price elasticity of demand for cigarettes using a a panel survey microdata from Nepal~~, where the prevalence and consumption pattern of cigarettes represent the South Asian context~~. ~~Second, we demonstrated the efficacy of Deaton's method in estimating the price elasticity of cigarettes, by utilizing the cross-sectional data regularly collected for poverty monitoring in Nepal and the other countries in the region. With these aims in mind, we applied Deaton's method to survey data from NHRVS (2016 to 2018) to estimate the price elasticity of demand for cigarettes cigarette in Nepal.~~ We found that the demand is inelastic as price elasticity is ~~ranges~~ from -0.79 to -0.37, with an average value of -0.58. ~~This indicates that the cigarette demand is relatively inelastic in Nepal. This also provides additional evidence that the surveys primarily designed to monitor poverty and living standards can provide useful insights for measuring the price elasticity of cigarettes, as suggested by Deaton(13).~~

The dataset we used for the analysis ensures that findings are comparable to the south Asian region. We compare the statistics reported by other related papers in south Asian and international context. The prevalence of cigarette consumption and expenditure patterns reported from our analyses are comparable to national statistics from the countries in the South

Asian region and other LMICs_. In our paper, 15 percent of households report non-zero expenses on cigarettes. In the South Asian region, prevalence estimates range from 4.5 percent in Bhutan to 18.7 percent in Maldives, with an average regional prevalence of 10 percent (18, 19, 30, 31, 33-36).. The expenditure share on cigarette consumption, however, shows varies to the less extent: 3.9% in India, 4.8% in Bangladesh, 5.4% in Bosnia and Herzegovina, 3% in Serbia, and 7.6% in Uganda. These comparisons indicate that the data and statistics we use are representative of national statistics and hence ensures external validity for the South Asian region.

The findings related to prevalence and expenditure patterns using data from Nepal exhibit consistency when compared with its South Asian Counterparts as well as other LMICs. Nepalese households incur non-zero spending are 15 percent, which is higher than the regional average of 10 percent, the statistics lie within the range from 4.5 percent in Bhutan to 18.7 percent in the Maldives (19). The prevalence reported elsewhere exhibits wide variation, ranging from 1.5 percent in Nigeria to 7 percent in Uganda and 40 percent in Bosnia and Herzegovina and Serbia (28, 29, 31-33). Expenditure shares (5.14 percent for Nepal), on the other hand, exhibit limited variation, from 3.9 percent in India to 7.6 percent in Uganda (18, 29, 33, 34). Likewise, the regression estimates for unit value (0.78) and budget share (0.0066) also fall within the wide range reported in the literature, which varies across the member nations in the region and other LMICs (14, 17, 28, 29, 31, 33, 35, 36). So far as price elasticity of demand is concerned, global evidence suggests wide variation, exhibiting both elastic and inelastic demands. For example, Vietnam (-0.165) and Zambia (-0.2) exhibit inelastic demand, while Pakistan (-1.06), rural Bangladesh (-1.38), and Bosnia and Herzegovina (-1.366) exhibit elastic demand. Most studies generally find inelastic demand for cigarettes (17, 33, 37, 39, 40, 41), but there is substantial cross-country variation in how smokers respond to changes in the price of cigarettes.

Formatted: Font: 12 pt

In the Deaton's method, the efficiency of the estimates relies on two factors: 1) how well the clusters' expenditure share and unit values capture the commonalities in the prices, and 2) the estimates of leading coefficients from unit value and budget share regression. We find that cluster fixed effects strongly determine the variation in unit values and expenditure share, and this ensures that there is a significant commonality in the data at the cluster level that can be exploited to produce the elasticity estimates. Similarly, the coefficients in the budget share and unit values regression align with the available literature in this area. The coefficient on total consumption for unit value regression is 0.78, and that with budget share regression is 0.0066. Studies applying Deaton's method in developing country context report a wide variation in the coefficients for the unit value and budget share regressions: 0.10 and 0.435 in India, 0.147 and 0.0168 in Bangladesh, 0.23 and -0.06 in Uganda, -0.1483 and -0.1453 in Nigeria, 0.06 and 0.004 in Bosnia; 0.11 and 0.002 in Serbia; 0.10 and -0.001 in South Africa, and 0.09 and -0.04 in Croatia (14, 17, 30, 31, 33, 35, 37, 38). These values suggest that our estimates of the coefficients are within the range reported in the literature.

In literature, the estimated value of the price elasticity of demand for cigarettes ranges from -0.165 in Vietnam to -1.37 in Bosnia and Herzegovina(35, 39). These studies used mixed approaches – time series and cross-sectional data. Studies using the time series method report the estimate in the inelastic range (0 to -1) and most of these studies belong to developed countries – the values range from -0.61 to -0.93(40). In developing countries, studies using time series method report a price elasticity of -0.165 in Vietnam and -0.2 in Zambia(41, 42). These figures indicate that consumers are evidently less responsive to the rise in cigarette prices. On the other hand, the studies using Deaton's method provide price elasticity estimates in both elastic and inelastic ranges. The majority of the countries report the estimate in an inelastic range – for example, -0.41 and -0.179 in rural and urban India, respectively, -0.89 in urban Bangladesh, -0.33 in Uganda, -0.639 in Serbia, and -0.620 in Nigeria(14, 17, 30, 31, 33).

On the other hand, a few studies report elasticities that are unitary elastic and even elastic – for example, -1.06 in Pakistan, -1.38 in rural Bangladesh, -1.366 in Bosnia and Herzegovina(17, 35, 43).

Policy relevance: Nepal's estimated value of price elasticity is relevant to the policymakers in the following ways. First, the estimates, together with their lower and upper limits, fall within the range of (-1, 0), suggesting that the cigarette consumption pattern in Nepal is relatively inelastic. This pattern aligns with the those reported in other countries in the South Asian region. This means that consumers reduce cigarette consumption by less thannearly 0.6 percent when the price increases by one percent. Therefore, a tax-increase policy on cigarettes, which raises the market prices, may reduce cigarette consumption to a lesser extent. This suggests that the government should combine the cigarette taxation policy with other measures (such as restrictions on smoking in public places, support for nicotine replacement therapies) to control cigarette consumption. Such a policy response can be expected in other settings where the demand for cigarette consumption is inelastic, such as in India and Bangladesh. Second, policymakers are also interested in the impact of taxation on cigarettes on revenue because it is directly affected by the consumer's demand response to price changes. Since the demand for cigarettes is relatively inelastic, an increase in excise tax could lead to higher revenue. With inelastic demand, consumers are less likely to reduce their consumption in response to a price increases, meaning that tax revenue could rise despite the higher prices of cigarettes. In Nepal, a recent policy discussion paper on excise tax on cigarettes in the 2024/25 budget examined the implications of tax increases on government revenue (22) The paper highlights that experience from the last three years demonstrates that increasing the tobacco taxes is an effective way to raise revenues.

Formatted: Font: Bold

Limitations: Our approach to estimating the price elasticity of demand for cigarettes is not without limitations. First, due to the low frequency of reported cigarette consumption, we used

Formatted: Font: Bold

repeated observations from a pool of three-year panel surveys to generate the price elasticity estimates. We corrected for the potential autocorrelation by allowing year-fixed effects into the regression equations. This approach prevents the possible bias. Second, unlike many other studies, we were unable to report disaggregated estimates of the price elasticity by policy relevant variables due to the relatively low frequency of positive cigarette consumption within each cluster. Despite these limitations, our analysis provides a robust estimate of price elasticity that align with the existing literature in the South Asian context and current practices in using price elasticity estimates in the policy context of Nepal.

Future research directions: Based on our analysis, the demand of cigarette smoking will benefit from Depending upon the data availability, it may be possible to extend this study, where researchers may consider heterogeneity of the sample population for analysis (for example, based on income levels, rural-urban settings, age groups, and gender). This has the advantage of making tax and tobacco control policies more targeted and fairer. It is also plausible to collect data at frequent intervals and examine the smokers' behaviour, since this will help make informed and timely policy and behaviour-related decisions. Likewise, new surveys available in the region comprise a broader set of questions related to tobacco and cigarette smoking. It may be meaningful to utilize the datasets from these surveys to examine and make inter- and intra-regional comparisons of the shifts in the behavioural patterns of the smokers.

Formatted: Font: Bold, Italic

Acknowledgment: We sincerely thank the World Bank and all the individuals involved in collecting and making the panel data available, which has been invaluable for our research.

Competing Interest: None declared.

Disclaimers**.** The views expressed in the submitted article are his or her own and not an official position of the institution or funder.

Funding/Support: The authors received no financial support for this research.

References

1. WHO. Tobacco use falling: WHO urges countries to invest in helping more people to quit tobacco2021 2024-12-16. Available from: https://www.who.int/news/item/16-11-2021-tobacco-use-falling-who-urges-countries-to-invest-in-helping-more-people-to-quit-tobacco?utm_source=chatgpt.com.

2. Shariful Islam M, Rashid M, Sizear MI, Hassan R, Rahman M, Parvez SM, et al. Cigarette smoking and associated factors among men in five South Asian countries: A pooled analysis of nationally representative surveys. Plos one. 2022;17(11):e0277758.

3. West R. Tobacco smoking: Health impact, prevalence, correlates and interventions. Psychology & health. 2017;32(8):1018-36.

4. Burki TK. WHO releases latest report on the global tobacco epidemic. The Lancet Oncology. 2021;22(9):1217.

5. Anh PTH, Ross H, Anh NQ, Linh BN, Minh NT. Direct and indirect costs of smoking in Vietnam. Tobacco control. 2016;25(1):96-100.

6. John RM, Sung H-Y, Max WB, Ross H. Counting 15 million more poor in India, thanks to tobacco. Tobacco control. 2011;20(5):349-52.

7. Bobak M, Jha P, Nguyen S, Jarvis M. Poverty and smoking. Oxford University Press; 2000.

8. Gupta PC, Puntambekar N, Assunta M. South Asia’s evolving tobacco hydra: moving from quandary to hope. Tobacco Control. 2022;31(2):142-5.

9. WHO. Framework Convention on Tobacco Control. World Health Organization; 2003 2024-12-16.

10. Gravely S, Giovino GA, Craig L, Commar A, D'Espaignet ET, Schotte K, Fong GT. Implementation of key demand-reduction measures of the WHO Framework Convention on

Tobacco Control and change in smoking prevalence in 126 countries: an association study. The Lancet Public Health. 2017;2(4):e166-e74.

11. Rijo M. John HR, Blecher E. A rational taxation system of bidis and cigarettes to reduce smoking deaths in India. Economics for Health. 2011.

12. Jha P, Khan J, Mishra S, Gupta P. Raising taxes key to accelerate tobacco control in South Asia. bmj. 2017;357.

13. Deaton A. Quality, Quantity, and Spatial Variation of Price. The American Economic Review. 1988;78(3):418-30.

14. John RM. Price elasticity estimates for tobacco products in India. Health Policy Plan. 2008;23(3):200-9.

15. Joseph RA, Chaloupka FJ. The influence of prices on youth tobacco use in India. nicotine & tobacco research. 2014;16(Suppl_1):S24-S9.

16. Selvaraj S, Srivastava S, Karan A. Price elasticity of tobacco products among economic classes in India, 2011–2012. BMJ open. 2015;5(12):e008180.

17. Ahmed N, Mozumder TA, Hassan MT, Huque R. Demand for tobacco products in Bangladesh. Tobacco Control. 2022;31(6):737-43.

18. Huque R, Abdullah SM, Hossain MN, Nargis N. Price elasticity of cigarette smoking in Bangladesh: evidence from the Global Adult Tobacco Surveys (GATS). Tob Control. 2024;33(Suppl 2):s51-s8.

19. WHO. Tobacco Retail Price Data 2024 [updated 2024-12-16. Available from: https://apps.who.int/gho/data/view.main.TOBRETAILv.

20. Economics for Health. South Asia Tobacco Control Report. 2022 2024-12-16.

21. Organization WH. Tobacco control: Raise taxes on tobacco2025. Available from: https://www.who.int/data/gho/data/themes/topics/raise-taxes-on-tobacco.

22. National Development Research Institute. Taxing Tobacco in Nepal: What can be done. National Development Research Institute; 2020.

23. National Development Research Institute. Policy Paper: Excise Tax on Cigarettes in the 2024/25 Budget. Kathmandu, Nepal: National Development Research Institute,; 2024.

24. Harris JE, López-Valcárcel BG. Asymmetric peer effects in the analysis of cigarette smoking among young people in the United States, 1992–1999. Journal of health economics. 2008;27(2):249-64.

25. Wickham H, Averick M, Bryan J, Chang W, McGowan LDA, François R, et al. Welcome to the Tidyverse. Journal of open source software. 2019;4(43):1686.

26. Berge L, Krantz S, McDermott G, Lenth R. Package 'fixest'. 2021.

27. Lumley T, Lumley MT. The survey package. hospital. 2007;24:1.

28. Canty A, Ripley B. Package 'boot'. Bootstrap Functions CRAN R Proj. 2017.

29. Walker T, Kawasoe Y, Shrestha J. Risk and vulnerability in Nepal: Findings from the household risk and vulnerability survey: World Bank; 2019.

30. Vladisavljevic M, Zubović J, Đukić M, Jovanović O. Tobacco price elasticity in Serbia: evidence from a middle-income country with high prevalence and low tobacco prices. Tobacco control. 2020;29(Suppl 5):s331-s6.

31. Chelwa G, van Walbeek C. Does cigarette demand respond to price increases in Uganda? Price elasticity estimates using the Uganda National Panel Survey and Deaton's method. BMJ open. 2019;9(3):e026150.

32. NRB. Current Macroeconomic and Financial Situation Tables. Nepal Rastra Bank; 2018 2024-12-16.

33. Adeniji F. Consumption function and price elasticity of tobacco demand in Nigeria. Tobacco Prevention & Cessation. 2019;5.

34. Perera KMN, Guruge GD, Jayawardana PL. Household expenditure on tobacco consumption in a poverty-stricken rural district in Sri Lanka. Asia Pacific Journal of Public Health. 2017;29(2):140-8.

35. Gligorić D, Pepić A, Petković S, Ateljević J, Vukojević B. Price elasticity of demand for cigarettes in Bosnia and Herzegovina: microdata analysis. Tobacco Control. 2020;29(Suppl 5):s304-s9.

36. NSO India. Household Consumption Expenditure Survey, 2022-2023. National Statistical Office, Government of India; 2023.

37. Dare C, Boachie MK, Tingum EN, Abdullah S, van Walbeek C. Estimating the price elasticity of demand for cigarettes in South Africa using the Deaton approach. BMJ open. 2021;11(12):e046279.

38. Barać ŽA, Burnać P, Rogošić A, Šodan S, Vuko T. Cigarette price elasticity in Croatia–analysis of household budget surveys. Journal of applied economics. 2021;24(1):318-28.

39. Viet Nguyen C, Le TT, Nguyen NH. The impact of cigarette prices on smoking participation and tobacco expenditure in Vietnam. Plos one. 2021;16(12):e0260415.

40. Kohler A, Vinci L, Mattli R. Cross-country and panel data estimates of the price elasticity of demand for cigarettes in Europe. BMJ open. 2023;13(6):e069970.

41. Nguyen N, Nguyen AN. Price elasticity estimates of cigarette demand in Vietnam using survey data. Tobacco Induced Diseases. 2021;19(1).

42. Stoklosa M, Goma F, Nargis N, Drope J, Chelwa G, Chisha Z, Fong GT. Price, tax and tobacco product substitution in Zambia: findings from the ITC Zambia Surveys. Tobacco Control. 2019;28(Suppl 1):s45-s52.

43. Nayab D, Nasir M, Memon JA, Khalid M, Hussain A. Estimating the price elasticity for cigarette and chewed tobacco in Pakistan: evidence from microlevel data. Tob Control. 2020;29(Suppl 5):s319-s25.

6. Supplementary Material or Appendices

Not Available

We thank the reviewers for reading our paper carefully and providing valuable comments that have been tremendously helpful in improving it. This document explains our response to each comment.

Reviewer #1:

Comment 1: *The introduction is lengthy and could be more concise, particularly in discussing regional comparisons. Streamline the introduction by condensing regional comparisons into a single paragraph.*

Response: Thank you very much for this feedback. We have reviewed the Introduction section carefully and made it more concise. We have condensed the regional comparisons. The changes can be found in the tracked-changes version of the revised manuscript.

Comment 2: *There is need to provide an overview of the trend of tobacco prices and/or excise tax in Nepal. This should be part of the Introduction section.*

Response: Thank you again for this important suggestion. We have added the following detail in the fourth paragraph of the introduction section.

Table 1 below shows the trend of cigarette prices and taxes in Nepal over the period of 10 years. The cigarette prices have been increasing at constant rate of ~1.00 USD (in PPP) between 2012 to 2022. This means that the household face similar increment in prices before and during the survey period. During the same period, the excise taxes have remained stable between 26 – 30 during the survey period.

Table 1: Cigarette price and tax in Nepal between 2012 and 2022.

	2012	*2014*	*2016*	*2018*	*2020*	*2022*
Retain price of cigarette per 20 sticks in PPP dollars	*3.65*	*4.69*	*5.63*	*6.78*	*8.38*	*10.43*

Taxes as percentage of cigarette price (%)	*29.86*	*27.79*	*26*	*30*	*27*	*31.39*
Source: https://www.who.int/data/gho/data/themes/topics/raise-taxes-on-tobacco						

Comment 3: *The numerator for Equation 7 should have a w bar, not an underlined w.*

Response: Thank you. We have corrected this.

Comment 4: *The discussion is lengthy and repetitive, particularly in comparing expenditure shares and coefficients across countries. Condense the comparison of expenditure shares and coefficients into a table for clarity and brevity.*

Response: Thank you for providing opportunity to revise the discussion. We have removed the repetitive points in the discussion and attempted to make the comparison condense. We have revised the section as follows.

The findings related to prevalence and expenditure patterns using data from Nepal exhibit consistency when compared with its South Asian Counterparts as well as other LMICs. While Nepalese households that incur non-zero spending are 15 percent, which is higher than the regional average of 10 percent, the statistics lie within the range from 4.5 percent in Bhutan to 18.7 percent in the Maldives (19). The prevalence reported elsewhere exhibits wide variation, ranging from 1.5 percent in Nigeria to 7 percent in Uganda and 40 percent in Bosnia and Herzegovina and Serbia (28, 29, 31-33). Expenditure shares (5.14 percent for Nepal), on the other hand, exhibit limited variation, from 3.9 percent in India to 7.6 percent in Uganda (18, 29, 33, 34). Likewise, the regression estimates for unit value (0.78) and budget share (0.0066) also fall within the wide range reported in the literature, which varies across the member nations in the region and other LMICs (14, 17, 28, 29, 31, 33, 35, 36). So far as price elasticity of demand is concerned, global evidence suggests wide variation, exhibiting both elastic and inelastic demands. For example, Vietnam (-0.165) and Zambia (-0.2) exhibit

inelastic demand, while Pakistan (-1.06), rural Bangladesh (-1.38), and Bosnia and Herzegovina (-1.366) exhibit elastic demand. Most studies generally find inelastic demand for cigarettes (17, 33, 37, 39, 40, 41), but there is substantial cross-country variation in how smokers respond to changes in the price of cigarettes.

Comment 5: *No mention of future research directions beyond the current limitations.*

Response: As suggested, we have identified areas of future research (See Discussion, Paragraphs 5) by adding following paragraph.

Depending upon the data availability, it may be possible to extend this study, where researchers may consider heterogeneity of the sample population for analysis (for example, based on income levels, rural-urban settings, age groups, and gender). This has the advantage of making tax and tobacco control policies more targeted and fairer. It is also plausible to collect data at frequent intervals and examine the smokers' behaviour, since this will help make informed and timely policy and behaviour-related decisions. Likewise, new surveys available in the region comprise a broader set of questions related to tobacco and cigarette smoking. It may be meaningful to utilize the datasets from these surveys to examine and make inter- and intra-regional comparisons of the shifts in the behavioural patterns of the smokers.

Comment 6: *The referencing should be reviewed. For instance, Walker et al. (2019)(27) should be Walker et al. (27). Also, note that there has to be space before an open bracket – review the whole manuscript.*

Response: Thank you. We have now corrected the references.

Reviewer #2:

Comment 1: *To be more specific Deaton's model is well suited for the cross-sectional data analysis and independent household observations. There seem to be some efforts from authors in this domain however are not well described and thus not possible to assess in the terms of their appropriateness and violating the main feature of current research in terms of replicability.*

Response: Thank you very much for providing us with an opportunity to elaborate on how the extension of Deaton's approach to panel data does provide useful results after reasonable assumptions and suitable adjustments. In the methodology section (specification and estimation, paragraph 3), we have added the following paragraphs.

In the pooled OLS set-up of equations 1 and 2, we adjust for possible bias that may arise from repeated measurements over three waves, possibly due to temporal trends in unit values and budget shares, temporal correlation in the error terms of the respective equations, and unobserved changes in cigarette consumption patterns that may be correlated with the covariates. We correct for these as follows:

1. *We add year-specific fixed effects to ensure that between-cluster variation in the unit value and budget share regressions is free from temporal trends. This ensures that the basic requirement to apply the Deaton demand model is maintained.*
2. *In these equations, within-cluster variation may be due to (i) variation in cigarette consumption preferences over the three-year period, (ii) autocorrelation in the error terms in equations 1 and 2, and (iii) measurement error (correlation between the error terms of equations 1 and 2). Of these, (iii) is key to Deaton's correction strategy for*

measurement error. We minimize bias that may result from (i) and (ii) with the following adjustments and assumptions:

- *We adjust for temporal correlation in u^1_{hct} and u^o_{hct} by allowing the errors to cluster over time while estimating the measurement error.*
- *We assume that policy-driven shifts in cigarette preferences are minimal during the survey period – a stable, marginal change in cigarette prices and taxes between 2016–2018 (shown in Table 1) provides evidence that this assumption is met.*
- *Cigarette consumption behaviour exhibits a stable pattern over short time interval. A one-year gap between each survey wave makes preference changes unlikely – longitudinal studies show that transitions between smoker and non-smoker states are rare within one year (24).*

Together, with these assumptions and adjustments, the coefficient estimates from the pooled OLS and measurement error corrections still maintain the properties of the original Deaton (1988) model.

Comment 2: *For robustness of the results, it would be preferable to use also all three waves of surveys to estimate elasticities separately and present results for those as further validation of the approach.*

Response: Yes, we have performed robustness checks in individual years. The estimate ranges -0.15 in 2016 to -0.95 in 2018, and both values indicate inelastic demand. These values are reported in the notes of Table 5 as follows.

We also undertake robustness checks of $\widehat{\varepsilon_p}$ across the three survey panels – the estimates range from -0.15 in 2016 to -0.95 in 2018.

Comment 3: *please do a thorough revision of the literature review as some of the information seem not to fit the contents of the papers cited. For instance, reference (14) to John (2008) indicate that for additional products to bidis and leaf tobacco elasticities are estimated, however the paper only investigates cigarettes, bidis and leaf tobacco.*

Response: Thank you very much for providing an opportunity to revise our literature review and discussion. We have thoroughly revised it, and it is available in the track changes in the text.
