# Supplementary material for: Price elasticity of demand for cigarettes in Nepal: Evidence from a lower middle-income country in South Asia using Deaton’s demand model
Source: PLoS One. 2026 Mar 30;21(3):e0345594. doi: 10.1371/journal.pone.0345594 (PMC13035141; doi:10.1371/journal.pone.0345594)
Supplement: Attachment — Submitted filename: Comments.docx [file pone.0345594.s001.docx]

**Reviewer 1 comments:**

**[Comment R1.1]** As I indicated previously, there should be space before the reference. For instance, from the first 6 lines of the fist paragraph of the introduction, it is evident that my previous comment was not considered:
Cigarette smoking is declining globally; however, its prevalence remains highest in South Asia. Recent evidence report that 27.1% of people aged 15 and above in South Asia smokes cigarettes, with Nepal reporting an even higher rate of 30.4%, compared to the global average of 22.3% (1, 2). Cigarette smoking is documented as one of the important modifiable riskfactors for leading non-communicable diseases(3, 4). Beyond its impact on health, smoking further aggravates living standards and poverty(5-7).

The authors should review the whole manuscript, accordingly.

**[Response]** Thank you. We have thoroughly revised the manuscript to address the reviewer’s suggestions on citation format.

**Reviewer 2 comments:**

The manuscript significantly improved on depth and quality of explaining the process. The use of proofreading service is strongly suggested. Some minor comments to be addressed are made in the text of the manuscript.

**[Response:]** Thank you for providing us an opportunity to clarify many of our statistical and conceptual approaches. We have thoroughly proofread the manuscript and made the suggested corrections. We have also addressed the comments made in the text of the manuscript.

**[Comment R2.1]** consult the consumption of cigarettes / tobacco in Balkan coutnries and the asia is only fraction of it, need to be amended

**[Response]** Thank you for your suggestion. We agree that cigarette smoking is very high in Balkan countries. However, we prepared this manuscript in the context of South Asia. Therefore, we did not mention any reference to Balkan countries in the opening sentence. However, we discussed the cigarette smoking behaviour and papers on elasticities from the Balkan countries in the discussion section (page no 18, paragraph 3, line 6 onward).

**[Comment R2.2]** suggest to reword the sentence to make it clearer if the reference to South Asia is related to Nepalese values or if South Asia is compared to the global values.

**[Response]** Thank you for pointing out the ambiguity. We have now revised the opening sentence to make it clear to the reader. We have made the following revision in page 4, para 1 line 2-5.

Cigarette smoking is declining globally; however, its prevalence remains highest in South Asia. Recent evidence reports that 27.1% of people aged 15 and above in South Asia smoke cigarettes—exceeding the global average of 22.3%—with Nepal reporting an even higher prevalence of 30.4% (1, 2).

**[Comment R2.3]** not necesserily given the conficdence interval of expenditure elasticity, suggest tomodificate the wording to eg more responsive to income growth

**[Response]** Thank you. We have changed the interpretation in the abstract and finding sections (page 2, abstract/results and page 17, last paragraph) of the document. These changes are available in the track changes.

**[Comment R2.4]** no need to manetion negatgive sign of the coefficient

**[Response]** Thank you. We have made the suggested corrections.

**[Comment R2.5]** brackets should be used across the paper for introducing the citations

**[Response]** Thank you. We have followed the recommended citation format for the Plos One.

**[Comment R2.6]** add also the line with NPR for reference to the survey based unit values in the analysis

**[Response]** Thank you. We have added the equivalent NPR values in page 6, Table 1, row 2.

| **Year** | **2012** | **2014** | **2016** | **2018** | **2020** | **2022** |
| --- | --- | --- | --- | --- | --- | --- |
| Retail price (per 20 sticks in PPP dollars) | 3.65 | 4.69 | 5.63 | 6.78 | 8.38 | 10.43 |
| Retail price (per 20 sticks in equivalent NPR^*^) | 95 | 130 | 176 | 211 | 266 | 342 |
| Taxes as percentage of cigarette price (%) | 29.86 | 27.79 | 26 | 30 | 27 | 31.39 |
| **Source**: The data was accessed from <https://www.who.int/data/gho/data/themes/topics/raise-taxes-on-tobacco> (23).  ^*^The PPP conversion factors for NPR were obtained from  <https://data.un.org/Data.aspx?d=WDI&f=Indicator_Code%3APA.NUS.PPP> (24) | | | | | | |

**[Comment R2.7]** it is not clear how this happens in the cluster-level setup where household level charactreristics are averaged out

**[Response]** the correlation between error terms of Equation 1 and 2, $\hat{\sigma}^{10},$ that appears in Equation 5 is derived from the panel data at household level regression. We corrected the autocorrelation issue when deriving the estimate of $\hat{\sigma}^{10}$, which is a panel regression. We have improved the sentence for clarity on this issue by modifying the sentence in page 10, line 17-19.

In this step, we account for correlation among households over time to address the autocorrelation in error terms ($\hat{\sigma}^{10})$ of Equation 1 and 2.

**[Comment R2.8]** suggest to elaborate also on the expenditure (often used as income proxy) elasticity as it also play crucial role in forming demand decision and subsequently budget revenues and related health implications

**[Response]** Thank you for the helpful comments. We have added the following lines in the policy relevance section (page: 19, section: policy relevance, line: 8-12; page: 20, line: 8-12)

However, a positive and unitary elastic expenditure elasticity suggests that rising incomes or increases in household expenditure can partially offset the price-induced reduction in cigarette demand, thereby moderating the overall decline in cigarette consumption and reinforcing the persistence of cigarette demand.

Here, the positive and unitary expenditure elasticity plays a complementary role by indicating how consumption responds to rising incomes. A positive expenditure elasticity suggests that economic growth or increases in household expenditure may offset some of the consumption-reducing effects of higher prices, thereby sustaining or even expanding the tax base.
