# Supplementary material for: Price elasticity of demand for cigarettes in Nepal: Evidence from a lower middle-income country in South Asia using Deaton’s demand model
Source: PLoS One. 2026 Mar 30;21(3):e0345594. doi: 10.1371/journal.pone.0345594 (PMC13035141; doi:10.1371/journal.pone.0345594)
Supplement: Attachment — Submitted filename: Response to Reviewers.docx [file pone.0345594.s002.docx]

We thank the reviewers for reading our paper carefully and providing valuable comments that have been tremendously helpful in improving it. This document explains our response to each comment.

**Reviewer #1:**

***Comment 1:*** *The introduction is lengthy and could be more concise, particularly in discussing regional comparisons. Streamline the introduction by condensing regional comparisons into a single paragraph.*

**Response:** Thank you very much for this feedback. We have reviewed the Introduction section carefully and made it more concise. We have condensed the regional comparisons. The changes can be found in the tracked-changes version of the revised manuscript.

***Comment 2:*** *There is need to provide an overview of the trend of tobacco prices and/or excise tax in Nepal. This should be part of the Introduction section.*

**Response:** Thank you again for this important suggestion. We have added the following detail in the fourth paragraph of the introduction section.

*Table 1 below shows the trend of cigarette prices and taxes in Nepal over the period of 10 years. The cigarette prices have been increasing at constant rate of ~1.00 USD (in PPP) between 2012 to 2022. This means that the household face similar increment in prices before and during the survey period. During the same period, the excise taxes have remained stable between 26 – 30 during the survey period.*

*Table 1: Cigarette price and tax in Nepal between 2012 and 2022.*

|  | *2012* | *2014* | *2016* | *2018* | *2020* | *2022* |
| --- | --- | --- | --- | --- | --- | --- |
| *Retain price of cigarette per 20 sticks in PPP dollars* | *3.65* | *4.69* | *5.63* | *6.78* | *8.38* | *10.43* |
| *Taxes as percentage of cigarette price (%)* | *29.86* | *27.79* | *26* | *30* | *27* | *31.39* |
| *Source: https://www.who.int/data/gho/data/themes/topics/raise-taxes-on-tobacco* | | | | | | |

***Comment 3:*** *The numerator for Equation 7 should have a w bar, not an underlined w.*

**Response:** Thank you. We have corrected this.

***Comment 4:*** *The discussion is lengthy and repetitive, particularly in comparing expenditure shares and coefficients across countries. Condense the comparison of expenditure shares and coefficients into a table for clarity and brevity.*

**Response:** Thank you for providing opportunity to revise the discussion. We have removed the repetitive points in the discussion and attempted to make the comparison condense. We have revised the section as follows.

*The findings related to prevalence and expenditure patterns using data from Nepal exhibit consistency when compared with its South Asian Counterparts as well as other LMICs. While Nepalese households that incur non-zero spending are 15 percent, which is higher than the regional average of 10 percent, the statistics lie within the range from 4.5 percent in Bhutan to 18.7 percent in the Maldives (19). The prevalence reported elsewhere exhibits wide variation, ranging from 1.5 percent in Nigeria to 7 percent in Uganda and 40 percent in Bosnia and Herzegovina and Serbia (28, 29, 31-33). Expenditure shares (5.14 percent for Nepal), on the other hand, exhibit limited variation, from 3.9 percent in India to 7.6 percent in Uganda (18, 29, 33, 34). Likewise, the regression estimates for unit value (0.78) and budget share (0.0066) also fall within the wide range reported in the literature, which varies across the member nations in the region and other LMICs (14, 17, 28, 29, 31, 33, 35, 36). So far as price elasticity of demand is concerned, global evidence suggests wide variation, exhibiting both elastic and inelastic demands. For example, Vietnam (-0.165) and Zambia (-0.2) exhibit inelastic demand, while Pakistan (-1.06), rural Bangladesh (-1.38), and Bosnia and Herzegovina (-1.366) exhibit elastic demand. Most studies generally find inelastic demand for cigarettes (17, 33, 37, 39, 40, 41), but there is substantial cross-country variation in how smokers respond to changes in the price of cigarettes.*

***Comment 5****: No mention of future research directions beyond the current limitations.*

**Response:** As suggested, we have identified areas of future research (See Discussion, Paragraphs 5) by adding following paragraph.

*Depending upon the data availability, it may be possible to extend this study, where researchers may consider heterogeneity of the sample population for analysis (for example, based on income levels, rural-urban settings, age groups, and gender). This has the advantage of making tax and tobacco control policies more targeted and fairer. It is also plausible to collect data at frequent intervals and examine the smokers’ behaviour, since this will help make informed and timely policy and behaviour-related decisions. Likewise, new surveys available in the region comprise a broader set of questions related to tobacco and cigarette smoking. It may be meaningful to utilize the datasets from these surveys to examine and make inter- and intra-regional comparisons of the shifts in the behavioural patterns of the smokers.*

***Comment 6:*** *The referencing should be reviewed. For instance, Walker et al. (2019)(27) should be Walker et al. (27). Also, note that there has to be space before an open bracket – review the whole manuscript.*

**Response:** Thank you. We have now corrected the references.

**Reviewer #2:**

***Comment 1:*** *To be more specific Deaton's model is well suited for the cross-sectional data analysis and independent household observations. There seem to be some efforts from authors in this domain however are not well described and thus not possible to assess in the terms of their appropriateness and violating the main feature of current research in terms of replicability.*

**Response:** Thank you very much for providing us with an opportunity to elaborate on how the extension of Deaton’s approach to panel data does provide useful results after reasonable assumptions and suitable adjustments. In the methodology section (specification and estimation, paragraph 3), we have added the following paragraphs.

*In the pooled OLS set-up of equations 1 and 2, we adjust for possible bias that may arise from repeated measurements over three waves, possibly due to temporal trends in unit values and budget shares, temporal correlation in the error terms of the respective equations, and unobserved changes in cigarette consumption patterns that may be correlated with the covariates. We correct for these as follows:*

1. *We add year-specific fixed effects to ensure that between-cluster variation in the unit value and budget share regressions is free from temporal trends. This ensures that the basic requirement to apply the Deaton demand model is maintained.*
2. *In these equations, within-cluster variation may be due to (i) variation in cigarette consumption preferences over the three-year period, (ii) autocorrelation in the error terms in equations 1 and 2, and (iii) measurement error (correlation between the error terms of equations 1 and 2). Of these, (iii) is key to Deaton’s correction strategy for measurement error. We minimize bias that may result from (i) and (ii) with the following adjustments and assumptions:*

- *We adjust for temporal correlation in* $u_{hct}^{1}$*and* $u_{hct}^{o}$*by allowing the errors to cluster over time while estimating the measurement error.*
- *We assume that policy-driven shifts in cigarette preferences are minimal during the survey period – a stable, marginal change in cigarette prices and taxes between 2016–2018 (shown in Table 1) provides evidence that this assumption is met.*
- *Cigarette consumption behaviour exhibits a stable pattern over short time interval. A one-year gap between each survey wave makes preference changes unlikely – longitudinal studies show that transitions between smoker and non-smoker states are rare within one year (24).*

*Together, with these assumptions and adjustments, the coefficient estimates from the pooled OLS and measurement error corrections still maintain the properties of the original Deaton (1988) model.*

***Comment 2:*** *For robustness of the results, it would be preferable to use also all three waves of surveys to estimate elasticities separately and present results for those as further validation of the approach.*

**Response:** Yes, we have performed robustness checks in individual years. The estimate ranges -0.15 in 2016 to -0.95 in 2018, and both values indicate inelastic demand. These values are reported in the notes of Table 5 as follows.

*We also undertake robustness checks of* $\hat{\varepsilon_{p}}$ *across the three survey panels – the estimates range from -0.15 in 2016 to -0.95 in 2018.*

***Comment 3:*** *please do a thorough revision of the literature review as some of the information seem not to fit the contents of the papers cited. For instance, reference (14) to John (2008) indicate that for additional products to bidis and leaf tobacco elasticities are estimated, however the paper only investigates cigarettes, bidis and leaf tobacco.*

**Response:** Thank you very much for providing an opportunity to revise our literature review and discussion. We have thoroughly revised it, and it is available in the track changes in the text.
